# Machine learning and artificial intelligence in type 2 diabetes prediction: a comprehensive 33-year bibliometric and literature analysis

**DOI:** 10.3389/fdgth.2025.1557467

**Published:** 2025-03-27

**Authors:** Mahreen Kiran, Ying Xie, Nasreen Anjum, Graham Ball, Barbara Pierscionek, Duncan Russell

**Affiliations:** ^1^ Faculty of Health, Medicine and Social Care, Anglia Ruskin University, Chelmsford, United Kingdom; ^2^ Faculty of Business and Management, Cranfield University School of Management, Cranfield, United Kingdom; ^3^ School of Computing, University of Portsmouth, Portsmouth, United Kingdom; ^4^ Medical Technology Research Centre, Anglia Ruskin University, Chelmsford, United Kingdom; ^5^ Ocado Technology, Hatfield, United Kingdom

**Keywords:** type 2 diabetes mellitus (T2DM), machine learning (ML), artificial intelligence (AI), bibliometric analysis, predictive models

## Abstract

**Background:**

Type 2 Diabetes Mellitus (T2DM) remains a critical global health challenge, necessitating robust predictive models to enable early detection and personalized interventions. This study presents a comprehensive bibliometric and systematic review of 33 years (1991-2024) of research on machine learning (ML) and artificial intelligence (AI) applications in T2DM prediction. It highlights the growing complexity of the field and identifies key trends, methodologies, and research gaps.

**Methods:**

A systematic methodology guided the literature selection process, starting with keyword identification using Term Frequency-Inverse Document Frequency (TF-IDF) and expert input. Based on these refined keywords, literature was systematically selected using PRISMA guidelines, resulting in a dataset of 2,351 articles from Web of Science and Scopus databases. Bibliometric analysis was performed on the entire selected dataset using tools such as VOSviewer and Bibliometrix, enabling thematic clustering, co-citation analysis, and network visualization. To assess the most impactful literature, a dual-criteria methodology combining relevance and impact scores was applied. Articles were qualitatively assessed on their alignment with T2DM prediction using a four-point relevance scale and quantitatively evaluated based on citation metrics normalized within subject, journal, and publication year. Articles scoring above a predefined threshold were selected for detailed review. The selected literature spans four time periods: 1991–2000, 2001–2010, 2011–2020, and 2021–2024.

**Results:**

The bibliometric findings reveal exponential growth in publications since 2010, with the USA and UK leading contributions, followed by emerging players like Singapore and India. Key thematic clusters include foundational ML techniques, epidemiological forecasting, predictive modelling, and clinical applications. Ensemble methods (e.g., Random Forest, Gradient Boosting) and deep learning models (e.g., Convolutional Neural Networks) dominate recent advancements. Literature analysis reveals that, early studies primarily used demographic and clinical variables, while recent efforts integrate genetic, lifestyle, and environmental predictors. Additionally, literature analysis highlights advances in integrating real-world datasets, emerging trends like federated learning, and explainability tools such as SHAP (SHapley Additive exPlanations) and LIME (Local Interpretable Model-agnostic Explanations).

**Conclusion:**

Future work should address gaps in generalizability, interdisciplinary T2DM prediction research, and psychosocial integration, while also focusing on clinically actionable solutions and real-world applicability to combat the growing diabetes epidemic effectively.

## 1 Introduction

Diabetes Mellitus is a chronic disease that has potentially fatal consequences if left undetected. It has the potential to lead to serious illness, such as kidney failure, sight loss and limb amputations and even fatal consequences if not detected and treated effectively (1). The disease has affected millions of people worldwide, and its prevalence is expected to surge in the future given that ageing and obesity are major risk factors and both are rising.

Diabetes can be broadly classified into two main types, namely Type 1 Mellitus (T1DM) and Type 2 Diabetes Mellitus (T2DM). T1DM is caused by the autoimmune destruction of insulin-producing pancreatic cells, leading to insulin deficiency and chronic hyperglycemia, usually affecting children or teenagers. T2DM is a chronic metabolic disorder with a number of causal factors, including genetic predisposition and lifestyle factors, such as poor diet, a lack of physical activity, high blood pressure, and obesity. While both T1DM and T2DM are serious conditions requiring ongoing management, T2DM is more common and is largely preventable with early detection and lifestyle interventions (2).

According to the latest estimates from the International Diabetes Federation (3), 537 million people worldwide had diabetes in 2021, and this number is projected to rise to 643 million by 2030 and 783 million by 2045. The same report predicts that 541 million adults worldwide are at increased risk of developing T2DM. The increasing prevalence of diabetes is a major public health concern and emphasizes the need for effective and smart prediction, prevention, and management strategies.

Machine learning (ML) models and artificial intelligence (AI) have great potential in developing personalized prediction systems for diabetes. Scientists have leveraged ML and data mining techniques in several research areas related to diabetes, including identifying diagnostic and predictive factors in diabetes development, predicting diabetes, analyzing diabetic complications, developing drugs and therapies for diabetes, and studying the impact of genetic and environmental factors on the onset and progression of diabetes (4). By analyzing vast amounts of diabetes-related data, ML models can transform raw information into invaluable knowledge, unlocking new avenues for more effective prognosis, diagnosis, and treatment of diabetes (5, 6).

In recent years, several survey articles have explored the use of ML models and AI in diabetes research. Some of these reviews have examined the application of ML tools across various diabetes-related domains (4). Others have taken a more targeted approach, focusing on specific areas such as diabetes detection (7, 8), diabetes management (9, 10), or diabetes prediction (11, 12). These reviews offer valuable insights into the application of ML and AI in diabetes management and prognosis.

However, with the recent surge in publications related to ML and AI in diabetes research, conducting a *bibliometric analysis* of the literature can provide several valuable insights, including publication trends, research hotspots, geographical distribution, collaboration networks, journal analysis, methodological trends, funding and support, thematic clusters, and gaps and opportunities. Therefore, several bibliometric studies have also been conducted in the diabetes field, focusing on various aspects and research domains. For instance, (13) conducted a bibliometric analysis of diabetes prediction (in general) using ML algorithms. This study focused on a 12-year period (2009–2020), provided a snapshot of recent trends, and emphasized publication trends while identifying the leading countries and journals. Another bibliometric study (14) examined the growth of literature in the field of diabetes (in general) by utilizing data from the MEDLINE database for the period 1995–2004. The study aimed to identify the core journals in this field during that time. The authors in (15) conducted a bibliometric analysis to identify, visualize, and characterize meta-analyses on diabetic foot ulcer research, focusing on treatment approaches, risk factor analysis, and economic evaluations. This study covered publications from 1999 to 2022, with data retrieved from the Web of Science (WoS) core collection database. The authors in (16) performed a bibliometric analysis of research papers published in the field of ML and deep learning (DL) techniques applied to diabetes research (in general) from 2000 to 2022 (22 years). The articles were categorized into detection, prediction, and management. This involved the statistical analysis of published literature to identify global research trends and networks, highlighting key countries, institutions, journals, articles, citations, and research topics.

Despite the valuable insights provided by these studies, several limitations highlight the need for further investigation.

• Firstly, many existing studies (13, 14, 16) tend to focus on diabetes as a whole, without distinguishing between T1DM and T2DM. These are distinct research areas with unique pathophysiologies, management strategies, and challenges, and a broad approach often overlooks the nuanced priorities specific to each type.
• Secondly, the variability in the time periods covered by these studies, with some focusing on relatively short durations, limits the ability to derive comprehensive longitudinal insights into the evolution of research trends.
• Thirdly, while robust bibliometric techniques are applied, these studies often lack detailed analysis of thematic clusters and the methodological advancements that have occurred over time, which are critical for understanding shifts in research paradigms.
• Lastly, there is a notable gap in the literature regarding targeted analyses of machine learning prediction models for T2DM, as much of the focus has been on complications or broader applications of machine learning in diabetes research. These gaps highlight the need for a more focused and nuanced approach in future bibliometric analyses.

To address these gaps, this research conducted a comprehensive bibliometric and literature analysis of the T2DM prediction research using ML and AI over a 33-year period (1991–2024). To the best of our knowledge, this is the first bibliometric and literature analysis focused on the prediction of T2DM using ML and AI techniques. We offer a historical perspective and trace the evolution of research methodologies, utilizing more advanced bibliometric tools such as Bibliometrics. Specifically, the study seeks to map the intellectual structure through co-citation network analysis, identify and analyze distinct thematic clusters, assess the contributions and centrality of different ML methodologies, and highlight the foundational and applied research in the field. Our analysis delves deeper into methodological evolution, datasets utilized, most influential key features to train the ML models, and future research directions, with a particular emphasis on interdisciplinary approaches and emerging technologies.

The objectives of this study are as follows:

1. **Publication trends, citation analysis, and global collaboration patterns**: To analyze publication and citation trends over time, and explore international collaboration patterns and key countries’ roles.
2. **Thematic clusters**: To identify thematic clusters in T2DM research using ML. Summarize each cluster’s focus and centrality, assess the impact of ML models on prediction accuracy.
3. **Foundational methods, datasets, and key predictors**: To evaluate the foundational methods, datasets and predominant predictors used in T2DM prediction research, particularly focusing on ML algorithms and their effectiveness.
4. **Research gaps, emerging trends, and future directions**: To identify research gaps and analyze emerging trends in methodologies for predicting T2DM over decades. Highlight challenges and suggest future research directions.

### 1.1 Research questions

This study aims to evaluate the current research landscape, assess the evolution and impact of ML models in T2DM research, and identify key trends and gaps in the literature. Specifically, this study will explore the following research questions.

1. How has the research landscape on T2DM prediction evolved in terms of publication frequency, citation metrics, and international collaborations from 1991 to 2024?
2. How do different ML methodologies and applications contribute to the various thematic clusters within the field of T2DM prediction research, and what are the intellectual connections and centralities among these clusters as revealed by co-citation network analysis?
3. How have ML models evolved in the prediction of T2DM, and what trends and methodologies have emerged over the different decades from 1991 to 2024 in terms of data sources, algorithms, and predictors?
4. What are the future areas of research and associated challenges?

### 1.2 Organization of the study

Our research study is organized as follows: Section 2 details the comprehensive approach employed to select and analyze the keywords used in this study. The detailed bibliometric analysis by analyzing author affiliations, citation counts, publication trends, and international collaboration patterns has been presented in Section 3. The discussion on the network analysis is presented in Section 4. Section 5 analyses ML applications in predicting T2DM from 1991 to 2024, divided into four eras: 1991–2000, 2001–2010, 2011–2020, and 2021–2024. Section 6 presents future directions and Section 7 concludes our study.

## 2 Methodology for the selection of keywords and literature

This section begins by outlining the comprehensive approach used to select and analyze the keywords relevant to this study, specifically addressing research question 1. Following this, research methodology employed to select the research articles based on the identified keywords is discussed.

### 2.1 Keyword selection and refinement

The keyword selection process began with an initial gathering of keywords, guided by input from domain experts. This input was then combined with Term Frequency-Inverse Document Frequency (TF-IDF) (17) to identify keywords that are both relevant and comprehensive for the bibliometric research. Word clouds were used to visually represent the selected keywords. Finally, a curated set of keywords was finalised for dataset extraction.

#### 2.1.1 Preliminary keyword screening

Table 1a shows the initial set of keywords that were generated through the solicitation of domain expertise, encompassing both broad and specific terms relevant to T2DM and predictive modelling. The keywords were divided into primary and secondary categories based on their relevance and importance to the study. Each primary keyword was systematically combined with every secondary keyword using logical “AND” and “OR” operators to ensure comprehensive coverage and relevancy in search queries. The terms included fundamental descriptors like Diabetes mellitus and Type 2 diabetes, as well as methodological keywords such as Machine learning, Logistic Regression (LR), and Deep learning, to name a few. Furthermore, to capture various permutations of crucial terms such as “prediction” and “predicting,” the “*” operator for truncation alongside root keywords has been employed.

**Table 1a:** Initial data search.

Primary keyword	Secondary keyword (OR)	WoS	Scopus
	Machine learning	543	180
	Data mining	219	252
	Neural network	184	265
	Digital twins	7	8
1. Diabet* Predict*	Deep learning	223	207
2. Type 2 Diabet* Predict*	Random forest	262	289
3. Diabetes Mellitus Predict*	Logistic regression	535	475
	Ensemble learning	4	76
	Boosting algorithm	10	60
	Decision tree	22	217
	Total no. of documents	2,009	2,029

Table 1b presents the refined set of articles specifically focusing on T2DM prediction using ML algorithms. This refinement process involved a careful review of the titles and abstracts of the initially identified articles. The criteria for selection were strictly based on the relevance to T2DM prediction and the application of machine learning techniques. Consequently, we identified 1,808 articles as our initial dataset of T2DM literature. By narrowing down the dataset through this rigorous screening process, we ensured that the articles included in this study were directly pertinent to our research objectives.

**Table 1b:** Selected articles for initial analysis.

Primary keyword	Secondary keyword (OR)	WoS	Scopus
	Machine learning	440	150
	Data mining	80	93
	Neural network	55	68
	Digital twins	3	4
1. Diabet* Predict*	Deep learning	115	70
2. Type 2 Diabet* Predict*	Random forest	105	95
3. Diabetes Mellitus Predict*	Logistic regression	201	189
	Ensemble learning	4	30
	Boosting algorithm	5	25
	Decision Tree	11	65
	**Total no. of documents**	**1019**	**789**

After establishing a foundational set of keywords, further refinement was performed using the TF-IDF algorithm on the preliminary keyword screening dataset. For more details, please refer to Appendix.

#### 2.1.2 Final keyword selection

During this phase, we curated a set of keywords for the ultimate extraction of articles from multiple In review databases. The chosen keywords, as presented in Table 1c, were selected with the overarching objectives of the literature review in mind, as outlined in the Section “Aims and Objectives of Study.” A threshold of 0.70 was set to guide the selection process, ensuring that only the most relevant and impactful keywords were included. For instance, foundational keywords such as Diabetes mellitus’ and Type2 diabetes’ were chosen to delineate the research domain, ensuring that the corpus reflected the specific disease focus. Acknowledging the diverse landscape of predictive analytics, we adopted an interdisciplinary strategy by integrating “Data mining” and “Logistic regression,” showcasing the fusion of statistical and computational domains. Introducing “learning algorithm” and “neural network” allowed us to encompass a wide range of algorithmic methodologies, spanning from traditional statistical techniques to innovative AI methods.

**Table 1c:** Finally selected set of keywords based on TF/IDF score.

Primary keyword	Secondary keyword (OR)	No. of articles
	Machine learning	580
	Risk factors	145
	Data mining	153
	Risk score	67
	Logistic regression	150
1. Diabet* Predict*	Deep learning	125
2. Type 2 Diabet* Predict*	Risk assessment	42
3. Diabet Mellitus Predict*	Decision tree	203
4. Diabetes risk predict*	Random forest	177
	Learning algorithm	145
	Neural network	93
	Artificial intelligence	15
	Gradient boosting	25
	Predict* model	431
	**Total no. of documents**	**2351**

The predictive emphasis of the analysis was enhanced with terms like “Risk factor,” “Risk score,” “Risk assessment,” and “Predictive Model,” directing attention to literature focusing on prognostic evaluation. Interestingly, clinical terms like “Risk factor” and “Risk score” carry considerable importance, surpassing the anticipated prominence of algorithm-related terms such as “Machine learning” and “Neural network.” This indicates that while advanced algorithms are vital, the core of diabetes prediction research lies in their integration with traditional clinical assessments. “Risk assessment” bridges these algorithmic and clinical aspects, underscoring the importance of evaluation in utilizing predictive analytics effectively. Notably, we combined “Predictive model” and “Prediction model,” both of which had significant TF-IDF scores, into a single keyword “Predict* model” to streamline our search and ensure comprehensive coverage of predictive modeling research. Additionally, although terms like “Diabetes dataset” and “Diabetes patient” met the threshold criteria, they were considered too generic and therefore excluded from the final keyword set. Instead, we opted for more specific terms to ensure the precision of the literature retrieved. However, “Risk prediction” given its relevance and specificity, was included as a primary keyword to capture studies focused on risk prediction and assessment in diabetes.

### 2.2 Literature selection and data collection

**Figure 1:**
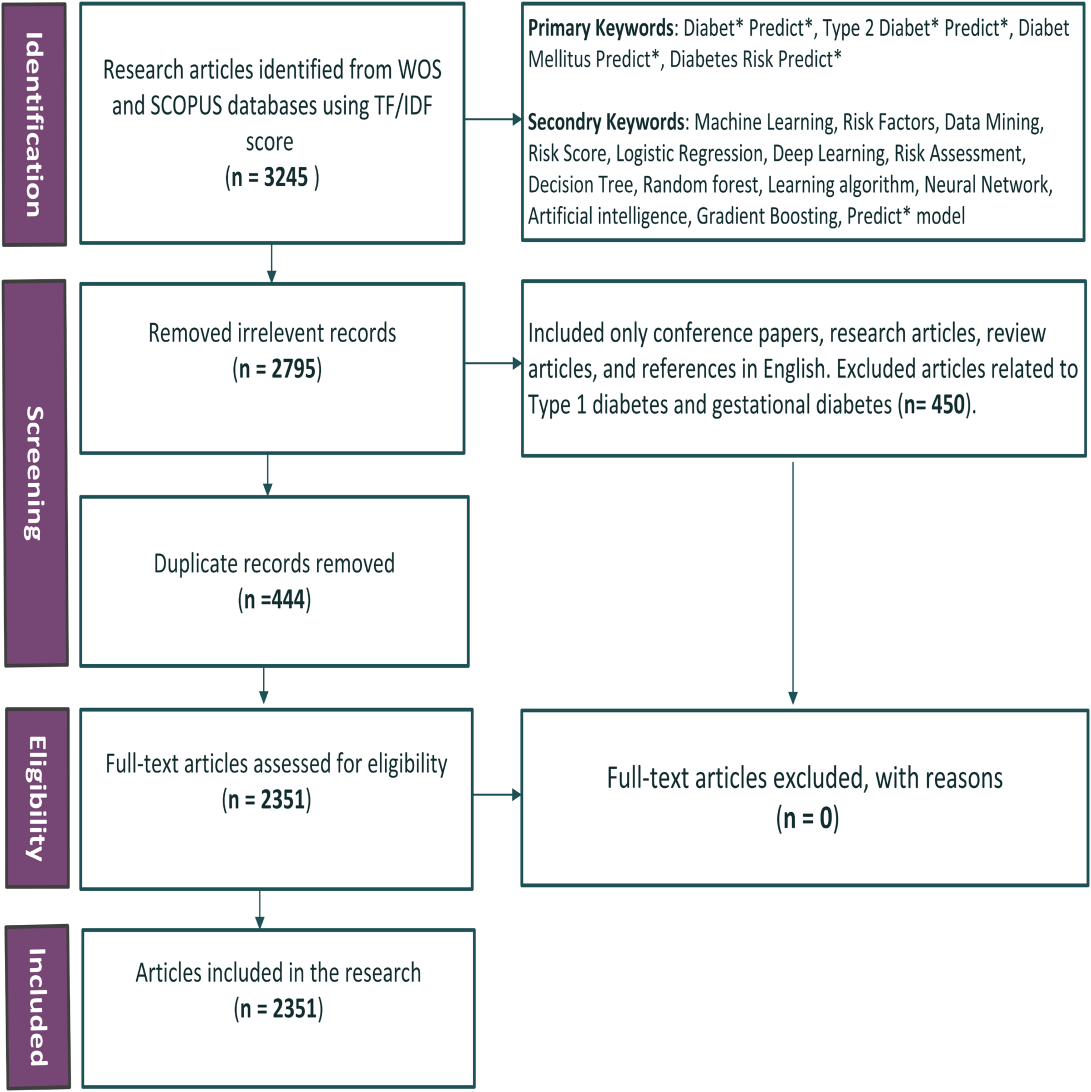
PRISMA flowchart for literature selection.

The process of literature selection and data collection was conducted systematically, following the PRISMA guidelines, and is detailed in the PRISMA flow diagram (Figure 1). Each step taken to refine the dataset is described below:

1. **Identification**: Articles were sourced from two comprehensive databases, Web of Science (WoS) and Scopus, to maximize coverage and minimize the exclusion of relevant studies. This broad scope reduced the risk of missing key literature due to database limitations. A finalized set of primary and secondary keywords, informed by expert input and refined through the TF-IDF method, guided the search. The refined results for the keyword sets are presented in Table 1c. The search targeted articles published between 1991 and 2024, relevant to T2DM prediction using ML techniques. This process initially identified 3,245 research articles. These articles were distributed equally among the five authors for review. Each author independently assessed their assigned articles using predefined relevance criteria to ensure consistency. Any discrepancies between reviewers were resolved through group discussions, ensuring objectivity and minimizing bias. Automation tools, including the Bibliometrix package in RStudio, were employed during the eligibility phase to identify and remove duplicate records. This automation streamlined the dataset, reducing it to 2,351 full-text articles for further analysis. The last search was conducted in August 2024. Bibliometric methods, including thematic clustering and co-citation network analysis, were applied to the refined dataset. Trends were assessed across four time periods: 1991–2000, 2001–2010, 2011–2020, and 2021–2024. Predictive models, datasets, and key variables were systematically evaluated during this process.
2. **Screening**: During the screening phase, irrelevant records were removed, reducing the dataset by 2,795 articles. Non-English articles, conference papers, and those unrelated to T2DM prediction were excluded. Further refinement removed records focusing on diagnosis, prevention, treatment, or complications of Type1 and gestational diabetes. 450 articles focusing on diagnosis, prevention, treatment or complications, Type1 and gestational diabetes were not included in the review.
3. **Eligibility**: After the initial screening, 444 duplicate records were identified and removed using the Bibliometrix tool in RStudio (18). This refinement left 2,351 full-text articles, which were assessed for eligibility.
4. **Inclusion**: Following the assessment, all 2,351 articles were deemed eligible and included in the research for further analysis. No articles were excluded at this stage.

## 3 Bibliometric analysis

To address research question 2 and to meet the objective of examining key attributes of diabetes prediction literature, this section presents a detailed bibliometric analysis. To visualize and map the literature database, we employed the Bibliometric package (18). Furthermore, the co-citation network, co-occurrence network, and collaboration network were graphically represented using the VOSViewer software (19). VOSViewer is a software tool used for constructing and visualizing bibliometric networks. These networks can include journals, researchers, or individual publications, and can be created based on citation, bibliographic coupling, co-citation, or co-authorship relations.

Analyzing citation counts, publication trends, and international collaborations provides insights into the evolution and impact of research in this field. As summarized in Table 2, the study spans 1991–2024, includes 1,115 sources, and reports an average of 12.09 citations per article. The authors in (20) highlighted the early 1990s as pivotal for advancements in AI and ML in healthcare, with researchers exploring these techniques for processing medical data, particularly for Type 1 and Type 2 diabetes. This study captures the evolution of AI and ML in diabetes management, from these early breakthroughs to recent advancements in 2024.

**Table 2:** Main information.

Main information about data	Results
Time Span	1991:2024
Sources (Journals, Books, etc)	1,115
Average citations per article	12.09
References	67,363
**Document contents**	
Keywords plus (ID)	4,289
Author’s keywords (DE)	67,363
Average citations per doc	12.09
References	67,363
**Document types**	
Article	1,728
Conference paper	321
Proceedings paper	186
Review	116

### 3.1 Analysis of research articles published annually w.r.t citations

**Figure 2:**
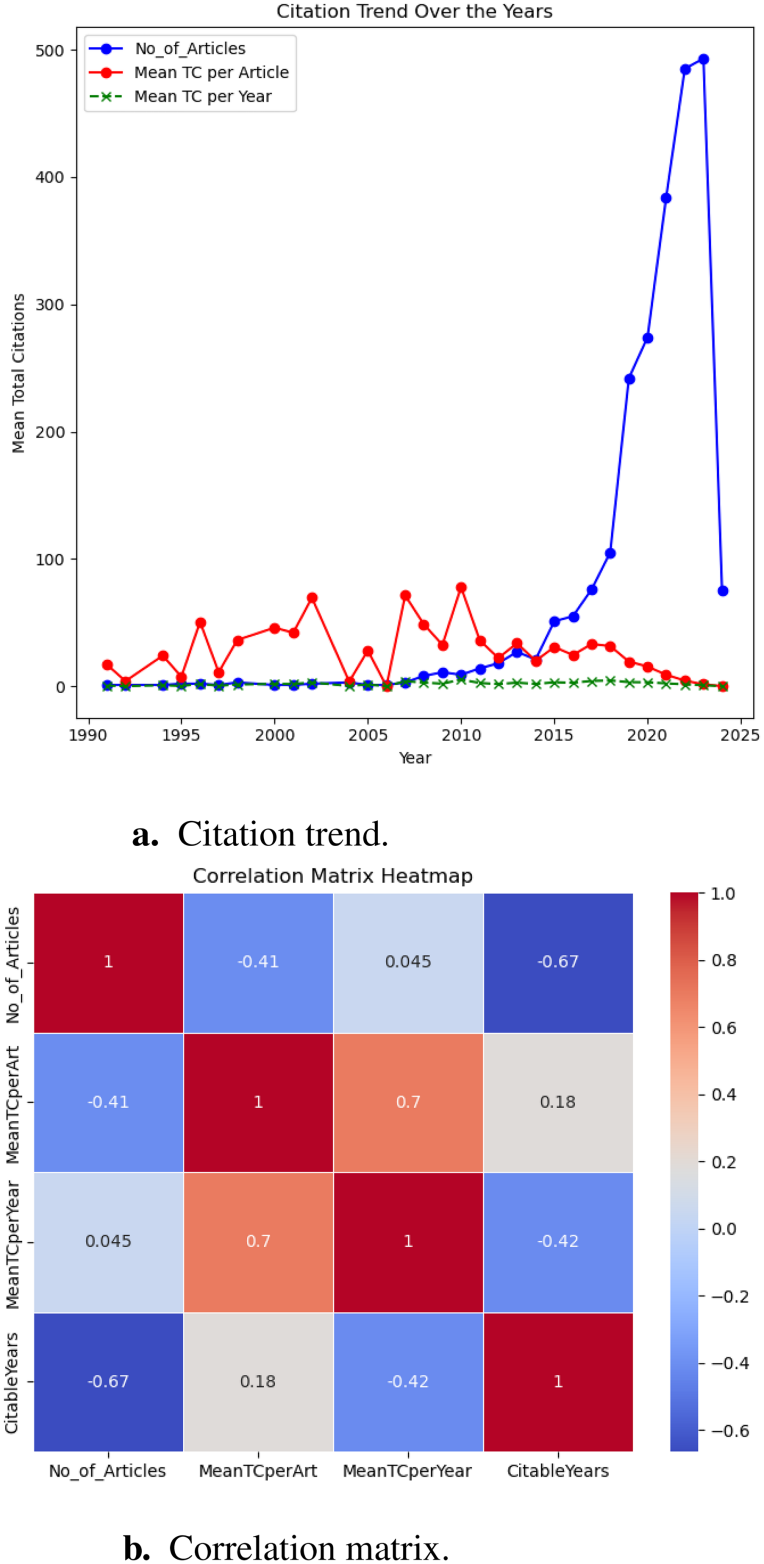
Analysis of research articles published annually w.r.t citations. (**a**) Citation trend. (**b**) Correlation matrix.

Our bibliometric analysis offers an in-depth view of how scholarly publication volume and citation metrics have evolved over time. As illustrated in Figure 2, we focused on two principal elements: the trends in citations across publication years (Figure 2a), and the temporal relationships among variables—including the number of articles published (No_of_Articles), the number of citable years (CitableYears), mean total citations per article (MeanTCperArt), and mean total citations per year (MeanTCperYear)—using a correlation matrix heatmap (Figure 2b).

1. **Exponential growth in research output and its consequences:** Post-2015, annual publication output surged from fewer than 50 articles in 2010 to over 450 by 2022 (Figure 2a). This surge aligns with growing interdisciplinary interest, increased funding, and AI advancements in healthcare. However, a negative correlation (r≈−0.41) between publication volume and mean citations per article (Figure 2b) suggests that as quantity increases, individual impact diminishes. Similar trends in AI-driven healthcare research indicate a focus on novelty over meaningful innovation, often resulting in redundant studies with incremental improvements (21–23). In T2DM prediction, frequent reuse of datasets like the UCI Pima Indians Diabetes Dataset has led to limited generalizability and reduced clinical relevance (4, 24). This lack of diverse population data restricts generalizability, clinical value, and translational impact, limiting the broader applicability of these predictive models (25). Without greater emphasis on real-world validation, and interdisciplinary collaboration, research risks stagnating in theoretical improvements rather than delivering meaningful advancements in healthcare.
2. **Citation trends: Research saturation and diminishing impact:** Despite the rapid rise in publications, the red line in Figure 2a (mean citations per article) fluctuates without sustained growth, while the green line (mean citations per year) remains relatively flat. This suggests inefficiencies in knowledge dissemination, where an expanding body of research does not necessarily translate into broader scientific progress. This suggests inefficient knowledge dissemination and “research saturation,” where publication proliferation does not equate to innovation (22). AI-driven T2DM prediction models often emphasize technical novelty over interpretability and validation, limiting clinical applicability (26). High-impact studies tend to integrate diverse data, explainable AI, and real-world implementation (27, 28). Addressing these gaps requires shifting research priorities from sheer publication volume to rigorously validated, clinically relevant work.
3. **Correlation matrix insights: The unequal distribution of research impact:** The correlation matrix (Figure 2b) reveals an uneven distribution of research influence, with a strong positive correlation (r≈0.7) between mean citations per year and mean citations per article. This indicates that a small subset of highly cited studies disproportionately impacts the field, while most publications contribute minimally. To address this imbalance, targeted investments should focus on high-quality, interdisciplinary research that integrates longitudinal datasets, diverse patient populations, explainable AI, and clinical validation to ensure real-world relevance (26). Without such efforts, the current trend risks further widening the gap between research volume and meaningful scientific advancements.
4. **Implications for researchers, journals, & policymakers:** These trends have significant implications for researchers, journals, and policymakers. Researchers often face mounting pressure to maintain high publication counts potentially reducing the time and resources devoted to deeper, more impactful investigations (29). It is also evidenced by the negative correlation between publication volume and mean citations per article. Journals, witnessing a marked influx of submissions, must strengthen peer-review standards and encourage practices such as data sharing, reproducibility, replication studies, and open-data initiatives (30). Policymakers and funding agencies should promote interdisciplinary collaborations and translational research to enhance the real-world impact of AI in healthcare. Encouraging global research partnerships and knowledge-sharing can facilitate the development of interoperable AI-driven health innovations. Recent bibliometric analyses highlight the growing prominence of AI in health informatics, underscoring the need for strategic investment in high-impact research that addresses emerging healthcare challenges (31). While numerous articles continue to appear each year, only a fraction yield novel insights, such as integrating real-world electronic health records (EHRs) or leveraging advanced deep learning architectures for precision risk stratification. Correspondingly, highly cited works in this space are often those that bridge multiple domains (e.g., endocrinology, computer science, bioinformatics) or that focus on interpretable AI to aid clinicians in practical decision-making. This underscores the broader theme that breakthrough research—encompassing originality, methodological rigour, and real-world utility—tends to have a more profound citation footprint and lasting impact on healthcare practice.

In summary, while AI-driven healthcare research continues to expand, bibliometric trends highlight the need to prioritize impactful studies over sheer publication volume. Addressing dataset limitations, ensuring clinical applicability, and fostering interdisciplinary collaboration are essential for meaningful progress. Insights from Figure 2 underscore the imperative to recalibrate research priorities. While the increasing volume of publications reflects strong engagement, citation data reveal gaps in quality and innovation. A collective effort among researchers, journals, and funding agencies is necessary to realign incentives toward interdisciplinary, impactful, and reproducible work. By doing so, the rapidly growing body of literature can drive tangible advancements in healthcare, particularly in T2DM prevention and management.

### 3.2 Analysis of literature w.r.t countries collaboration

**Figure 3:**
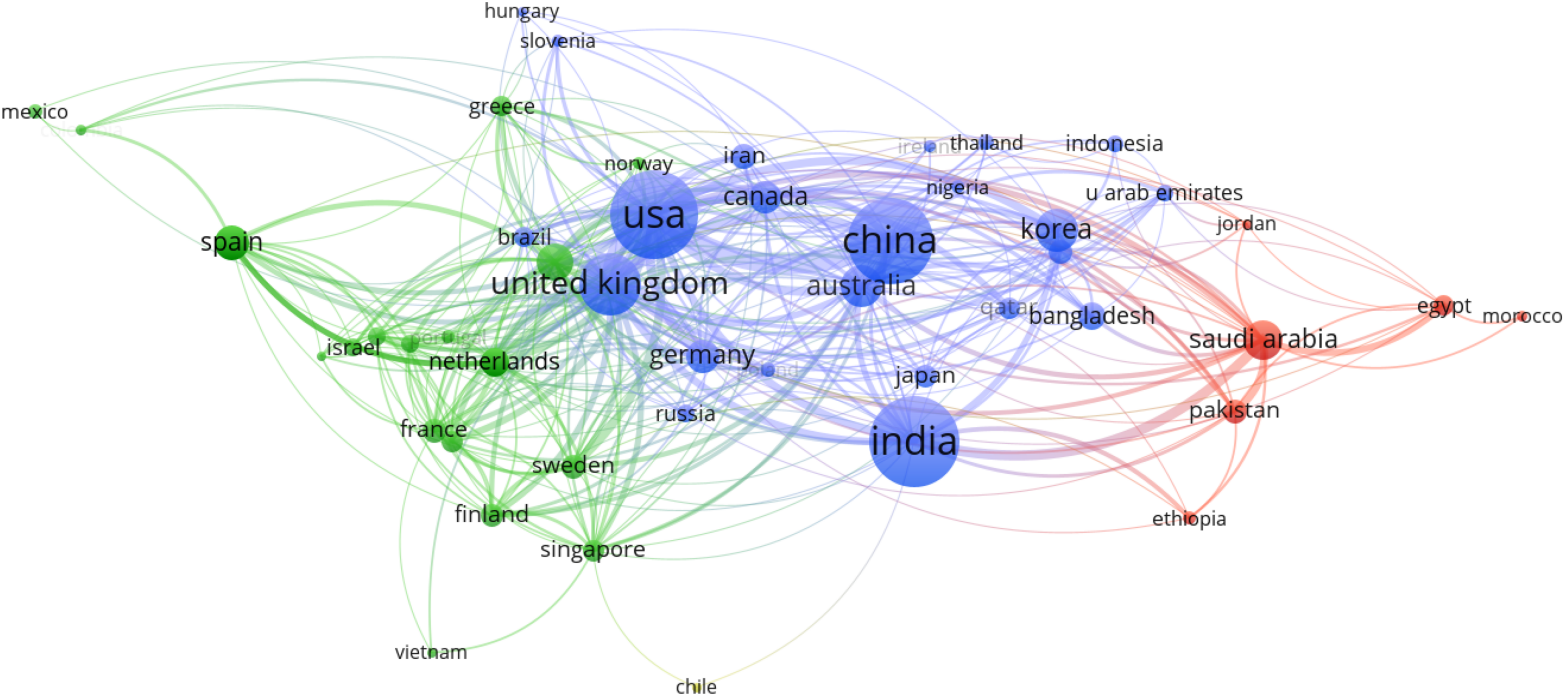
Network visualization of collaborative countries.

In this study, we employ a network analysis to explore the patterns of association between various countries. Figure 3 shows the visual representation of the network created using VOSviewer, which facilitates the comprehensive examination and interpretation of complex datasets. This visualization allows us to discern clusters and relationships among countries, providing a foundation for deeper analysis. Table 3 complements the visual insights gained from the network analysis by presenting quantitative metrics for the top five nodes within each cluster. These metrics include bridging centrality, closeness, and PageRank, which offer valuable insights into the roles and influence of individual countries within the collaboration network.

**Table 3:** Summary of collaborative network analysis.

Node	Cluster	Bridging	Closeness	PageRank
Saudi Arabia	1	69.17871495	0.013157895	0.040544105
Egypt	1	9.36403046	0.011494253	0.013054708
Pakistan	1	1.557900522	0.011764706	0.017074653
Jordan	1	0.193981938	0.010989011	0.006885467
Ethiopia	1	0.07269145	0.010526316	0.007911609
Italy	2	24.86787083	0.014925373	0.03789288
Singapore	2	23.87286495	0.013513514	0.024286976
Netherlands	2	8.96448097	0.01369863	0.029513399
France	2	7.742682939	0.013333333	0.022753563
Sweden	2	6.581808632	0.013157895	0.025261636
United Kingdom	3	226.996643	0.018181818	0.094932694
USA	3	225.9362586	0.017241379	0.116435363
China	3	83.62188851	0.015151515	0.057392505
India	3	55.15719477	0.014084507	0.043440715
Australia	3	53.29505222	0.015384615	0.045629771

Bridging centrality measures a node’s role as a bridge between different parts of the network. A higher bridging centrality indicates that a country acts as a key connector or conduit through which interactions between other countries occur. Closeness centrality reflects the average distance of a node to all other nodes in the network. A country with high closeness centrality can be interpreted as having direct and short paths to other nodes, indicating a potential for swift and efficient interactions. PageRank is a measure of node importance, which considers not only the quantity of connections but also the quality, as connections from more significant nodes carry more weight. In this context, a country with a high PageRank is seen as influential within the network, likely contributing to or benefiting from robust interactions.

It can be observed from Figure 3 and Table 3 that Cluster-1 comprised of countries such as Saudi Arabia, Egypt, Pakistan, and others, demonstrating moderate levels of bridging centrality. These countries appear to act as mediators, facilitating connections between other countries in the network. However, their closeness and PageRank values were relatively lower, suggesting a more peripheral role in the broader collaboration landscape. Cluster-2 encompassed countries like Spain, Italy, Netherlands, and Singapore, among others. Italy emerged as a notable influencer within this cluster, boasting the highest PageRank score. Meanwhile, Singapore and the Netherlands exhibited significant bridging centrality, indicating their pivotal roles in linking various countries within the collaboration network. The cohesive nature of this cluster suggests a tight-knit group of countries with strong collaborative ties, potentially focusing on prediction.

Cluster-3 included leading nations such as the USA, China, India, and others, each wielding considerable influence and connectivity within the collaboration network. The USA and the UK stood out with the highest PageRank values, underscoring their dominant positions in global collaboration networks. These countries play crucial roles in shaping research agendas, driving innovation, and fostering international partnerships across diverse fields. Overall, the analysis of international collaborations highlights the roles of key countries and regions, indicating a robust and interconnected global research network. Countries such as the USA and the UK continue to lead in terms of influence and collaboration, while emerging contributors like India and Singapore show the expanding geographical scope of impactful research.

## 4 Network analysis

**Figure 4:**
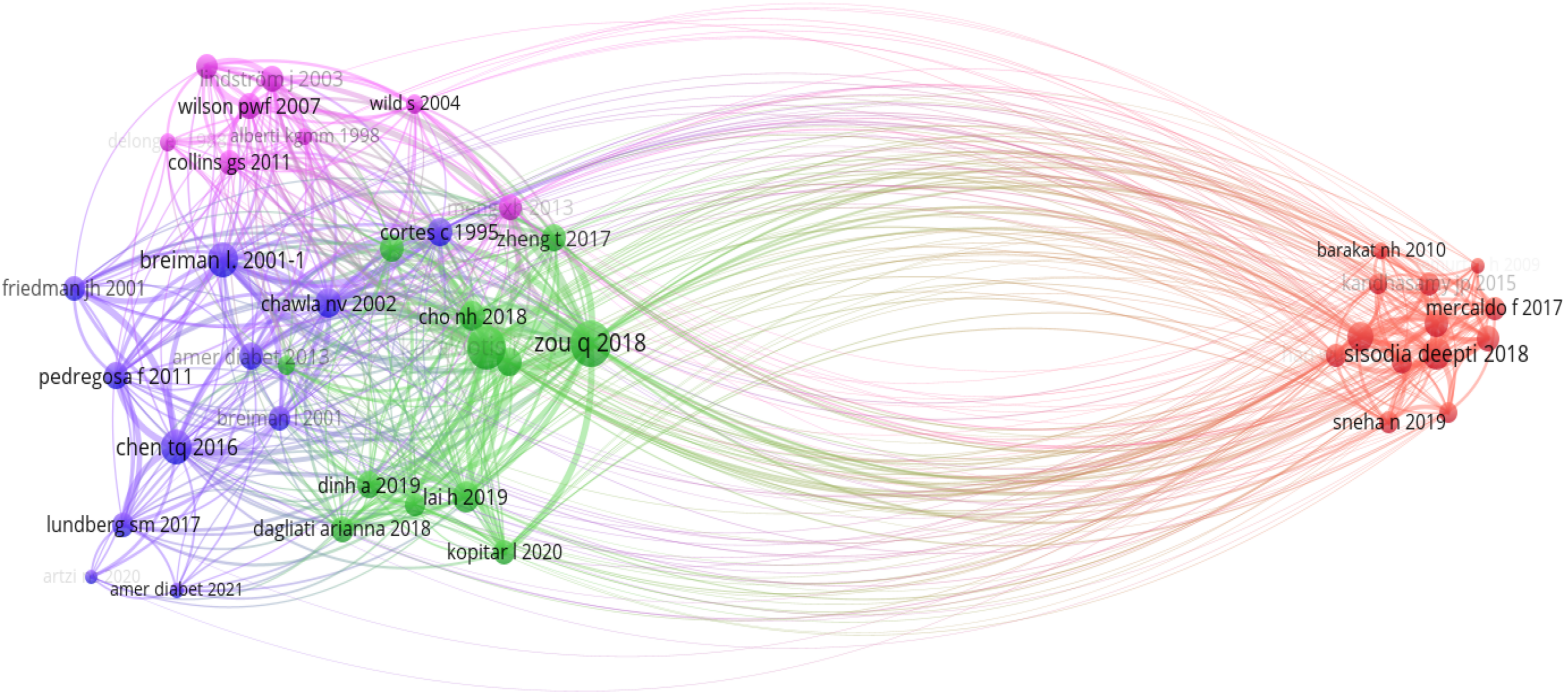
Co-citation network analysis.

Bibliometric methods for network analysis have proven to be effective tools for revealing both well-established and novel research topics. In this section, we utilized co-citation network analysis, a bibliometric method, to establish intellectual connections between significant research papers and map the intellectual structure of diabetes prediction research. This method focuses on the relationship or interaction between two publications and provides an overview of publications that have been cited together in other research articles. When two or more articles are cited together more frequently in other research articles, the probability of similarity between them is higher (32). As illustrated in our co-citation network analysis (Figure 4), there are four distinct clusters, each representing a unique aspect of the intersection between ML and diabetes studies: the blue, red, pink, and green clusters. These clusters were analyzed based on their thematic focus, as well as their bridging and closeness centrality measures within the co-citation network, providing insights into their roles and interconnections within the broader research landscape. A detailed summary of each cluster, including their thematic focus and centrality measures within the co-citation network, is provided in Table 4.

• **Foundational ML & statistical methodologies (Blue cluster):** In the context of our co-citation analysis, the blue cluster represents foundational ML and statistical methodologies that are fundamental to the advancement of diabetes research. This cluster incorporates seminal works that have contributed to the development and refinement of algorithms, particularly addressing prevalent issues in data science such as imbalanced datasets, exemplified by the work of (33) on the Synthetic Minority Over-sampling Technique (SMOTE). Furthermore, it includes references to widely-used tools such as sci-kit-learn, which have democratized the application of ML through their ease of access and versatility (34). It can be observed that there is a moderate closeness centrality observed in the blue cluster. It suggests that the methodologies it encompasses are broadly relevant to a wide array of studies within diabetes research. This relevance is attributed to the universal nature of foundational ML techniques, which are applicable across various subdomains, from basic biological research to clinical applications. Such techniques are often necessary prerequisites for more advanced, specialized research and provide a common language for scientists across disciplines. However, the cluster’s lower bridging centrality reveals a nuanced role. This could suggest that, although these foundational methods are widely used within individual research domains, they are less often at the forefront of interdisciplinary integrations that link disparate strands of diabetes research.
• **Epidemiological forecasts & lifestyle interventions (Pink cluster):** Encompassing a wide range of studies, the pink cluster explores epidemiological forecasts, lifestyle interventions, and the integration of digital health technologies in managing diabetes. It reflects a broader clinical and public health perspective, focusing on risk assessments [e.g., (35, 36)] and the global burden of diabetes (37), all of which are essential for a comprehensive understanding of diabetes management and prevention. The high closeness centrality associated with the pink cluster’s themes within the bibliometric network underlines the integral role these studies play in the broader scope of diabetes research. The research contained within this cluster lays the groundwork for various other domains within diabetes research, making it foundational. It offers essential insights into public health strategies, the development of clinical practices, and the formulation of healthcare policies.
• **Application of ML in predictive modeling (Green cluster):** In the green cluster of the co-citation network analysis, the central theme encapsulates the application of ML techniques to enhance the predictive modelling and analysis in diabetes research [e.g., (38, 39)]. Key findings within this cluster reveal that decision trees (DT), random forests (RF), neural networks (NN), and Gradient Boosting Machines (GBM) are particularly effective in predicting the onset of diabetes mellitus using a range of clinical and demographic data [e.g., (40, 41)]. Comparative studies within the cluster suggest that the more sophisticated ML models do not always yield clinically relevant enhancements over traditional regression models (42). This is critical as it underscores the need for carefully considering the choice of a prediction model in practical settings. Moreover, it can be observed that the green cluster demonstrates a high closeness centrality, which indicates their significant linkage within the research network. This closeness centrality is due to the cluster’s contribution to predictive health informatics, an area of heightened importance that harnesses ML techniques to foresee diabetes onset and progression. Such predictions are crucial for planning public health interventions and managing resources in healthcare systems. Moreover, the moderate bridging centrality of this cluster reveals its role in blending methodological advancements from machine learning with practical, actionable insights for clinical and epidemiological purposes.
• **Application of ML in clinical diabetes research (Red cluster):** The red cluster specifically targets the application of ML techniques for diagnosing, predicting, and managing diabetes. It includes innovative uses of algorithms for clinical data analysis aiming to enhance patient care and outcomes [e.g., (43, 44)]. A notable feature of the red cluster is its high centrality from the methodological (blue), public health-oriented (pink), and epidemiological (green) research clusters. The divergence is partly due to the unique data and specialized patient information required for clinical studies, which contrasts with the population-level data prevalent in public health and epidemiology studies. Moreover, the red cluster embodies the intersection of ML with clinical medicine, a path that is often separate from the public health and foundational research trajectories due to differing methodologies, publication cultures, and terminologies. The regulatory and ethical landscape governing clinical research further contributes to this separation, as these considerations demand stringent adherence to privacy and safety standards, which may not align with the broader ML research cited by the other clusters. Additionally, clinical application research is often driven by the immediacy of patient-centred outcomes and the rapid development and deployment cycle of medical technologies, creating a focused body of literature that prioritizes efficacy and safety. This patient-centric approach is less likely to interlace with the exploratory or predictive nature of the research found in the remaining clusters. Consequently, the red cluster’s progression forms a distinct branch within the research landscape, signalling a need for more concerted interdisciplinary efforts to bridge the gap and foster a more cohesive dialogue between these crucial areas of diabetes research.

**Table 4:** Summary of network analysis.

Cluster	Thematic focus	Key contributions	Closeness centrality	Bridging centrality
Blue	Foundational ML and statistical methods	– Development of algorithms for imbalanced datasets & tools for enhancing ML access such as SMOTE & sci-kit-learn	Moderate	Low
Pink	Epidemiological forecasts, lifestyle interventions	– Risk assessments, global burden of diabetes, integration of digital health technologies in management	High	Medium
Green	Application of ML in predictive modeling	– Effective use of ML algorithms such as DT, RF, NN, and gradient boosting machines for diabetes prediction	High	Moderate
Red	Application of ML in clinical diabetes research	– Use of algorithms for clinical data analysis, enhancing patient care and outcomes	Moderate	High

**Overall summary:** Our bibliometric analysis highlights the multi-faceted nature of ML research in diabetes, spanning foundational algorithm development, clinical studies, public health analyses, and predictive modeling. The thematic clusters underscore interconnected efforts to leverage ML for better understanding, predicting, and managing diabetes. Foundational methodologies (blue cluster) offer adaptable tools, public health insights (pink cluster) inform large-scale prevention strategies, predictive modeling advancements (green cluster) enable robust applications, and clinical applications (red cluster) translate advancements into patient-centered outcomes. Future research should prioritize interdisciplinary collaboration to bridge gaps between clusters, integrating methodologies, clinical insights, and public health strategies. Addressing regulatory and ethical challenges will be key to real-world implementation. The continued evolution of these clusters promises advancements in diabetes prediction research, improving prevention, diagnosis, and management globally.

## 5 Analysis of ML and AI models for T2DM prediction: a literature review (1991–2024)

This section addresses research question 3 of how ML models have evolved in the prediction of T2DM and complies with the objectives of evaluating foundational methodologies and statistical techniques, identifying gaps, and analyzing emerging trends from 1991 to 2024. It thoroughly reviews the models and techniques used over different decades, evaluates predominant predictors, assesses the impact of datasets on model accuracy, and highlights challenges and future research directions.

**Figure 5:**
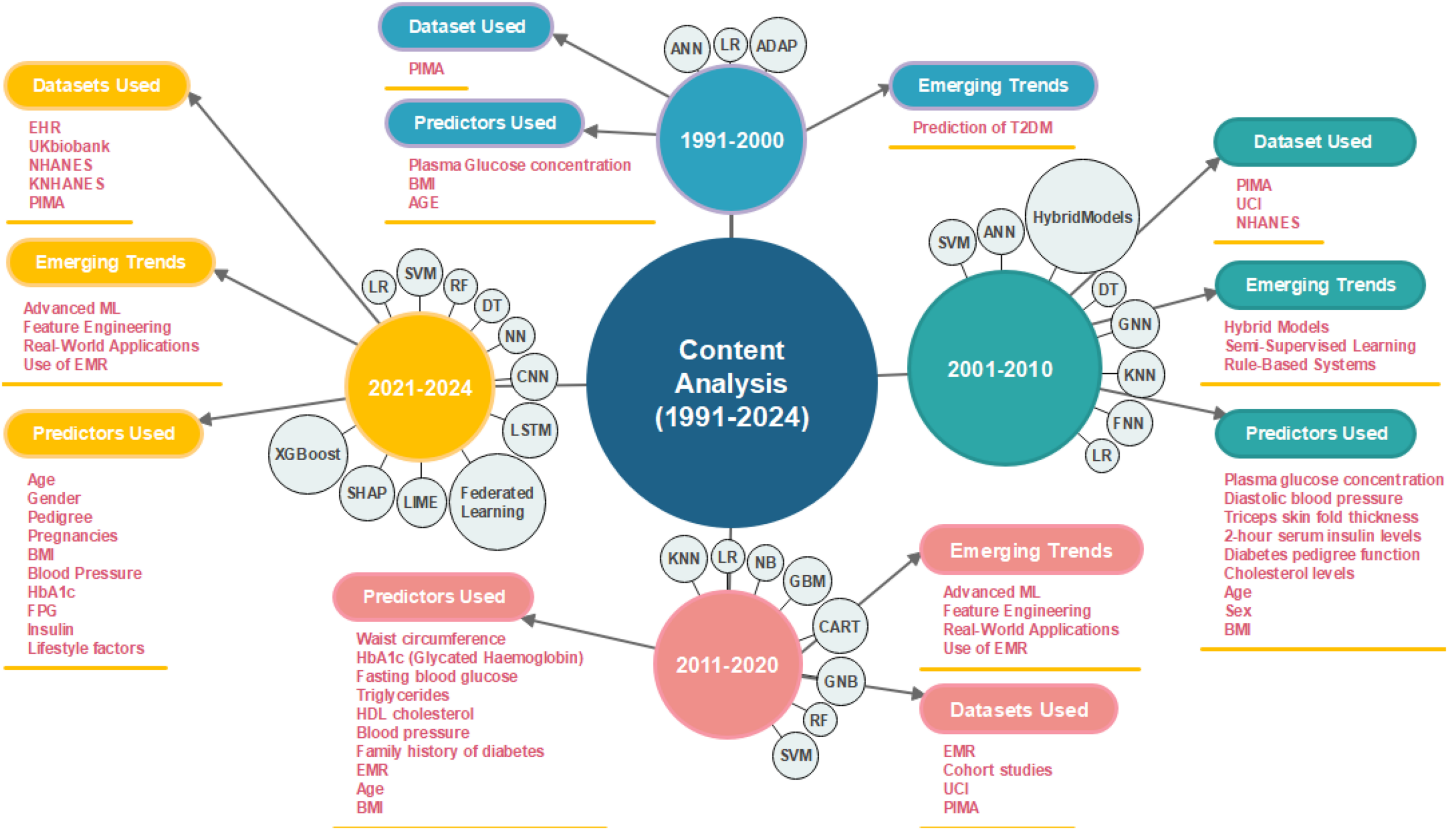
Content analysis and discussion of ML models for T2DM prediction (1991–2024).

This content analysis focuses specifically on the application of ML in predicting T2DMs. For the reader’s convenience, we analyzed the content for each decade, starting from 1991 to 2024, divided into the following eras: 1991–2000, 2001–2010, 2011–2020, and 2021–2024. For each era, we provide literature and discussion on (i) the ML models used for T2DM prediction, (ii) the datasets utilized to train the ML models, (iii) the predictors used, and (iv) emerging trends or topics in that era. Figure 5 provides a visual summary of these key elements for each era.

### 5.1 Methodology for the selection of literature

We follow the strategy outlined by Marcus et al. (45) for conducting a literature analysis on our curated dataset of 2,351 publications, utilizing a systematic and structured approach of TF-IDF (Section 2). To ensure a comprehensive analysis, we developed a systematic four-point scale for both qualitative assessment (relevancy score) and quantitative assessment (impact score). Two reviewers were selected to implement this methodology. Each reviewer independently assessed articles based on predefined relevance criteria. Any discrepancies in their ratings were resolved through discussion until a consensus was reached. This process helped maintain objectivity and minimize bias in our relevance scoring. The relevance score assesses how closely an article aligns with the specific focus of the study.

This has been determined based on the following criteria, scored on a four-point scale.

1. **Score 4 (Highly relevant):** Articles focused exclusively on ML algorithms for T2DM prediction, providing detailed analyses, results, and discussions.
2. **Score 3 (Moderately relevant):** Articles discussing ML for health outcomes, including T2DM, but not exclusively focused on T2DM prediction.
3. **Score 2 (Slightly relevant):** Articles addressing ML broadly or T2DM without specifically using ML for prediction.
4. **Score 1 (Not relevant):** Articles mentioning ML or T2DM only tangentially, with minimal relevance to the core topic.

After the qualitative assessment, articles that were deemed relevant underwent a quantitative assessment through impact score. The impact score evaluates the quantitative influence of an article, typically based on citation metrics and the article’s reach within the scientific community. We first obtained the total number of citations for each article from databases such as Google Scholar, Scopus, and Web of Science. Then to ensure a fair comparison, we normalized the citation counts. This involved calculating the average number of citations for similar articles (considering subject, journal, and publication year).^1^ Each article was assigned an impact score based on its citation count compared to the normalized average, using a quartile-based system to quantitatively assess its relative impact.

1. **Score 4 (Highly influential):** This score is assigned to articles in the top 25% (Q1 quartile) of citation counts, indicating they have the highest influence.
2. **Score 3 (Influential):** Articles in the second quartile (25%–50%) of citation counts receive this score. These articles have a high influence but fall below the top 25%.
3. **Score 2 (Average influence):** Assigned to articles in the third quartile (50%-75%) of citation counts, representing an average level of influence.
4. **Score 1 (Below average influence):** This score is given to articles in the bottom 25% (Q4 quartile) of citation counts, indicating they have the lowest influence among the set.

The overall score for each article was determined by summing its relevance and impact scores. To prioritize articles for detailed review, we averaged the combined relevance and impact scores from each rater. Articles with an average score exceeding a predetermined threshold of 3.5 were included in the in-depth analysis phase. This dual-criteria approach, integrating qualitative relevance assessment with quantitative impact analysis, ensures that the selected articles are both highly relevant and impactful in the field of T2DM prediction using ML. This methodology provides a comprehensive and systematic approach to identifying and analyzing the most significant research articles in this domain.

### 5.2 Literature analysis for time period 1991–2000

This era has been predominantly focused on the utilization of ML algorithms and existing technologies for the management of Type 1 diabetes, particularly in predicting and controlling blood glucose levels. For instance, (46–49).

In contrast, little attention has been paid to the prediction of T2DM, with only one study (50) employing ML algorithms to predict this condition. The study (50) evaluates the efficacy of Artificial Neural Networks (ANNs) in predicting diabetes and compares its performance with LR and the ADAP (Adaptative Perceptron) learning algorithm. Utilizing the Pima Indian diabetes dataset (51), which includes 768 cases with eight significant predictor variables (e.g., number of times pregnant, plasma glucose concentration, etc), the study identifies plasma glucose concentration, Body Mass Index (BMI), and age as the best predictors through a backward-elimination, stepwise approach. The NN model with one hidden node outperformed LR and ADAP, achieving a training classification accuracy of 77.43% and a test classification accuracy of 81.25%, compared to LR’s 77.60% and 79.17%, and ADAP’s 76% test accuracy.

### 5.3 Literature analysis for time period (2001–2010)

In comparison to the initial era (1991–2000), several studies conducted during 2001–2010, have focused on T2DM using various ML algorithms, datasets, and predictive features. Based on our developed systematic four-point scale for qualitative and quantitative assessment, we selected 18 highly influential articles published during this era.

**ML algorithms utilized:** During this period, several ML models were explored for T2DM prediction. Prominent models include Support Vector Machines (SVM), ANN, Hybrid Models, Semi-supervised Learning Models, DT (C4.5 Algorithm), General Regression Neural Networks (GRNN), K-means Clustering (KNN), LR, Fuzzy Neural Networks (FNN), Rule-based Methods such as Sequential Covering Approach (SQRex-SVM), and Eclectic Method for Rule Extraction. These algorithms reflect a variety of approaches, from classification and regression techniques to clustering and rule-based methods, highlighting the breadth of ML applications in T2DM prediction research during this period.

**Datasets utilised:** Several key datasets were utilized during this period, each contributing to the robustness of the research findings. Pima Indians Diabetes Dataset was extensively utilized for its detailed clinical features relevant to diabetes prediction. It was referenced in studies like (52–62) etc., highlighting its importance in ML research for diabetes. UCI Irvin ML Repository (63) was another frequently used dataset, supporting various studies such as (56, 64–66). This repository’s diverse datasets facilitated a broad range of ML applications. National Health and Nutrition Examination Survey (NHANES) provided a comprehensive set of health-related data, which was leveraged in the (67) for the prediction of T2DM study, illustrating its utility in large-scale health data analysis.

**Predominant predictors used for training ML models:** During this era, the predominant predictors used for training ML models in T2DM prediction research were primarily clinical and demographic variables. Clinical predictors such as BMI, plasma glucose concentration, diastolic blood pressure, triceps skin fold thickness, 2-hour serum insulin levels, diabetes pedigree function, and cholesterol levels were frequently employed due to their strong association with diabetes risk. Demographic predictors, particularly age and sex, were also commonly used, reflecting their significant impact on the likelihood of developing T2DM. These predictors were integral to many studies, including those leveraging datasets from the UCI Machine Learning Repository and the Pima Indians Diabetes Dataset.

In contrast, less emphasis was placed on lifestyle predictors such as physical activity, diet, smoking status, alcohol consumption, and exercise habits. Although these factors are recognized as influential in diabetes development, they were not as prominently featured in the predictive models of this period. Only a few studies, such as (67) integrated these lifestyle factors into their analysis. Additionally, genetic predictors like Single Nucleotide Polymorphisms (SNPs) were used in only one study (68), likely due to the limited availability of comprehensive genetic data during this timeframe. The focus on traditional clinical and demographic predictors reflects the research priorities and data availability of the era, while the relative underuse of lifestyle and genetic factors indicates areas for future exploration and integration in predictive modelling.

**Emerging trends:** This era saw innovative trends in T2DM prediction, including hybrid models, semi-supervised learning, and rule-based systems to enhance interoperability.

Hybrid models combine multiple algorithms to leverage the strengths of each and improve predictive performance. This approach helps in addressing the limitations of individual algorithms and enhances the robustness of the predictive models. For instance, the authors in (69) used the Simple KNN Algorithm for initial data validation and the C4.5 Algorithm for building the final classifier contributed to the robustness and high performance of the HPM, making it a reliable tool for predicting the incidence of T2DM in newly diagnosed patients. The study (70) applied SVM with rule extraction techniques to improve model interoperability and validate the model using a real-life dataset to ensure high prediction accuracy, sensitivity, and specificity. The paper (56) developed a hybrid neural network system that combines ANNs and FNN for the classification and prediction of T2DM. The primary objective is to increase the classification accuracy of medical data by integrating both fuzzy and crisp data and to evaluate the performance of this proposed hybrid system. The study (71) focuses on enhancing prediction accuracy by integrating fuzzy logic and NN techniques to model the complex relationships in diabetes data. The objectives are to demonstrate the effectiveness of this hybrid approach and to compare its performance with traditional ML methods.

Semi-supervised learning methods incorporate both labelled and unlabeled data into the model training process. This approach is particularly useful when labelled data is scarce or expensive to obtain, as it allows the model to learn from a larger dataset that includes unlabeled data. The author in (57) employs a semi-supervised learning method known as Laplacian SVM (LapSVM), that integrates labelled data with a significant amount of unlabeled data, leveraging the latter to improve the learning process. The model achieved higher accuracy and better generalization by utilizing the additional information from unlabeled data. Another study (57), applied General Regression Neural Networks (GRNN) for diabetes prediction and also incorporated semi-supervised learning algorithms. The use of GRNN, combined with semi-supervised learning, allowed the model to leverage the additional information provided by the unlabeled data, resulting in enhanced performance.

### 5.4 Literature analysis for time period (2011–2020)

Based on our developed systematic four-point scale, we selected and reviewed 55 highly influential articles published to predict T2DM using various ML models. In comparison to the 2001–2010 time period, the period from 2011 to 2020 has been transformative for T2DM prediction. The adoption of advanced ML algorithms, the expansion of feature sets, and the emphasis on real-world applicability represent the key emerging trends.

**ML algorithms utilized:** The most commonly utilized ML algorithms across these studies are LR, RF, SVM, Naïve Bayes (NB), kNN, GBM, Classification and Regression Trees (CART), and Gaussian Naïve Bayes. LR has been widely used across multiple studies due to its simplicity, effectiveness, and performance in binary classification problems (72–76). In comparative studies, RF has often outperformed other algorithms such as LR, KNN, and Gaussian Naïve Bayes (42, 73, 75, 76). It has been successfully applied across various datasets, including electronic medical records (EMR) and large-scale cohort studies (73, 76). SVM has been utilized in studies dealing with high-dimensional data spaces (77–80). While SVMs have shown competitive results, ensemble methods such as RF and GBM have often surpassed SVM in predictive accuracy (75, 76, 78, 79, 81, 82). Naïve Bayes is frequently utilized due to its simplicity and efficiency in handling large datasets [e.g., (75)]. KNN has been employed in studies emphasizing interpretability and simplicity (77, 80). However, KNN typically yields lower performance compared to more complex algorithms. GBM has gained popularity due to its high accuracy. Studies have utilized GBM and achieved high AUCs, with boosting techniques significantly improving model accuracy (76, 79). Classification and Regression Trees were often used in conjunction with other algorithms or as part of ensemble methods like RF (73, 76). While providing solid baseline performance, CART’s results were generally enhanced when used within ensemble methods. Gaussian Naïve Bayes has been employed in scenarios requiring probabilistic interpretation of predictions (73).

**Datasets utilized:** Similar to 2001–2010 era, among the most commonly used datasets is the Pima Indians Diabetes Dataset (75, 83–87). Additionally, the Henan Rural Cohort Study is used [e.g., (76)], providing valuable data from a large population sample in rural China. Furthermore, many studies leverage routinely collected EHR data from multiple health centres, offering rich, real-world insights into patient health metrics and outcomes (77, 80, 88).

**Predominant predictors used for training ML models:** Numerous studies in this era have focused on identifying demographic information such as age and gender, alongside medical conditions like hypertension and dyslipidemia, and lifestyle factors including physical activity and diet. Anthropometric measures like BMI and waist circumference, along with blood parameters such as glycated haemoglobin (A1c), fasting plasma glucose, triglycerides, and cholesterol levels, are commonly used to assess T2DM risk. Studies [e.g., (73, 89–91)] highlight that older age, high BMI, increased waist circumference, and a family history of diabetes are strong T2DM indicators. Incorporating HbA1c into predictive models has demonstrated high accuracy in identifying at-risk individuals (74, 92, 93). Additionally, lipid profiles, particularly elevated triglycerides and low HDL cholesterol levels, are significant predictors commonly associated with insulin resistance, a precursor to T2DM (90, 94).

Despite substantial advancements in research, there remain notable deficiencies, particularly in the integration of detailed dietary habits and nutritional patterns. Incorporating these variables could yield valuable insights into their impact on diabetes risk. Additionally, investigating genetic predispositions and their interactions with lifestyle and environmental factors could enhance the comprehensiveness of risk assessments. Furthermore, examining psychosocial factors, such as stress and mental health, could provide a more holistic understanding of diabetes development and management. Addressing these underrepresented areas in future studies will refine predictive models and contribute to a more thorough understanding of diabetes risk factors, ultimately improving prevention and management strategies.

**Emerging trends:** This period witnessed a notable transition in diabetes prediction research. Especially 2011–2016 was marked by a shift towards the utilization of ML tools and the exploration of diverse predictive variables. We observed three different research groups during this time period. The first group includes studies that utilize traditional clinical diabetes risk prediction techniques, which focus on large cohorts, but employ limited feature sets, such as (73, 95). The second group focuses on comparing ML models by utilizing classical diabetes risk factors as features, as demonstrated in (96, 97). The third group of related work considers a broader set of features that can be utilized to predict various diseases, such as (74). Additionally, during this time period, there was a trend of comparing different ML algorithms [e.g., (98, 99)] and identifying risk scores for variables associated with diabetes prediction [e.g., (5, 90)]. However, very few studies belonged to group 3. Furthermore, most studies were not generalizable to other populations, and handling missing values in large datasets was not frequently addressed. It is worth noting that the use of EMR for diabetes prediction dates back to 2012 (73).

From 2017 to 2020, researchers increasingly turned to more sophisticated algorithms and larger datasets to enhance the accuracy of diabetes prediction. For example, in (81), hidden patterns were extracted from data to anticipate outcomes for diabetes classification. In (100) and (101), fuzzy rules were generated using different methods for diabetes prediction. Additionally, researchers [e.g., (76, 101)] are working to identify novel optimal features such as urine and sweet taste that can aid in diabetes prediction, beyond basic features like age, gender, and BMI. We also observed a growing trend in utilizing socio-demographic and clinical/laboratory attributes (39, 76) and addressing issues such as missing data in predictive modelling, as evidenced in studies such as (102) and (103). While many authors have reported accuracy rates exceeding 85%, the majority of these studies have not been validated on populations with different race/ethnicity, and most of them have only used a limited number of features. Therefore, it is uncertain whether these models can be generalized to a larger population and how they will perform when more features are incorporated.

A critical trend observed is the diversity in the datasets utilized for developing these predictive models. The scope expanded to include a broader range of features, including biochemical markers, lifestyle factors, and even genetic data. This comprehensive approach has allowed for more accurate and personalized risk assessments. For instance, some studies incorporated electronic medical records and claims data, which provided a richer context and improved the models’ ability to predict diabetes onset at a population level.

The emphasis on feature engineering and the extraction of detailed features has been another notable trend. Researchers have extensively identified and validated a variety of predictive features. This granular approach has significantly enhanced the predictive power of the models. Moreover, the use of ensemble methods and hybrid models has become prevalent, combining multiple algorithms to leverage their strengths and mitigate individual weaknesses. The decade also saw a growing interest in real-world applications of these predictive models. Several studies (73, 104, 105) aimed to develop tools that could be integrated into clinical practice, enabling healthcare providers to identify high-risk individuals early and tailor preventive strategies accordingly.

In summary, these developments promise to enhance the accuracy and utility of predictive models, ultimately improving outcomes for individuals at risk of T2DM. Moreover, ensemble methods like RF and GBM were commonly the top performers across various studies, highlighting their robustness and high accuracy. LR continues to be a reliable benchmark model due to its simplicity and interpretability. Many studies emphasized the importance of feature selection and engineering, significantly impacting the performance of the models.

### 5.5 Literature analysis for time period (2021–2024)

Based on our developed systematic four-point scale strategy, we reviewed 65 highly influential papers published during this era. Researchers have increasingly combined advanced technologies such as medical devices, wearable and sensor technologies with ML, deep learning, and AI approaches to forecast T2DM (106–109). Additionally, similar to the 2011-2020 era, there is continued emphasis on identifying novel and effective non-invasive features that can assist in predicting T2DM (109–114).

**ML Algorithms utilized:** Analysis from 2021–2024 reveals that RF, SVM, XGBoost, and KNN remain popular (115–125). In contrast to 2001–2020, researchers focused on deep learning models, particularly NN (e.g., (126, 127), Convolutional Neural Networks (CNNs) [e.g., (128, 129)], and Long Short-Term Memory (LSTM) networks [e.g., (129, 130)], have become more prevalent due to their superior performance in handling large and complex datasets. Federated learning has emerged as a promising approach for collaborative research, allowing models to be trained on decentralized data sources without compromising patient privacy (131, 132). This technique facilitates large-scale, multi-centre studies and enhances the robustness of predictive models. Moreover, there is an increasing emphasis on the interpretability and explainability of ML models. Techniques such as SHAP (SHapley Additive exPlanations) (133) and LIME (Local Interpretable Model-agnostic Explanations) (134) are being used to help clinicians understand and trust the predictions made by these complex models (135, 136). The use of hybrid models and transfer learning is also on the rise.

**Dataset utilized:** Pima Indian Diabetes Dataset and EHR are still often cited due to their comprehensive features relevant to diabetes prediction (122, 126, 137). Additionally, EHR are widely used, providing real-world data essential for developing and validating predictive models (122, 126, 137). Other significant datasets include UK Biobank (117, 138, 139) extensive health-related data that supports robust predictive analysis. Although minimal studies found to be using NHANES and Korean National Health and Nutrition Examination Survey (KNHANES) dataset (126, 140, 141). A notable pattern in the use of these datasets is their application in cohort studies, highlighting their value in longitudinal research that tracks health outcomes over time (142–144). Additionally, many studies leverage real-world data and databases, indicating a trend towards using diverse and large-scale data sources to enhance the accuracy and generalizability of predictive models (138, 145).

**Figure 6:**
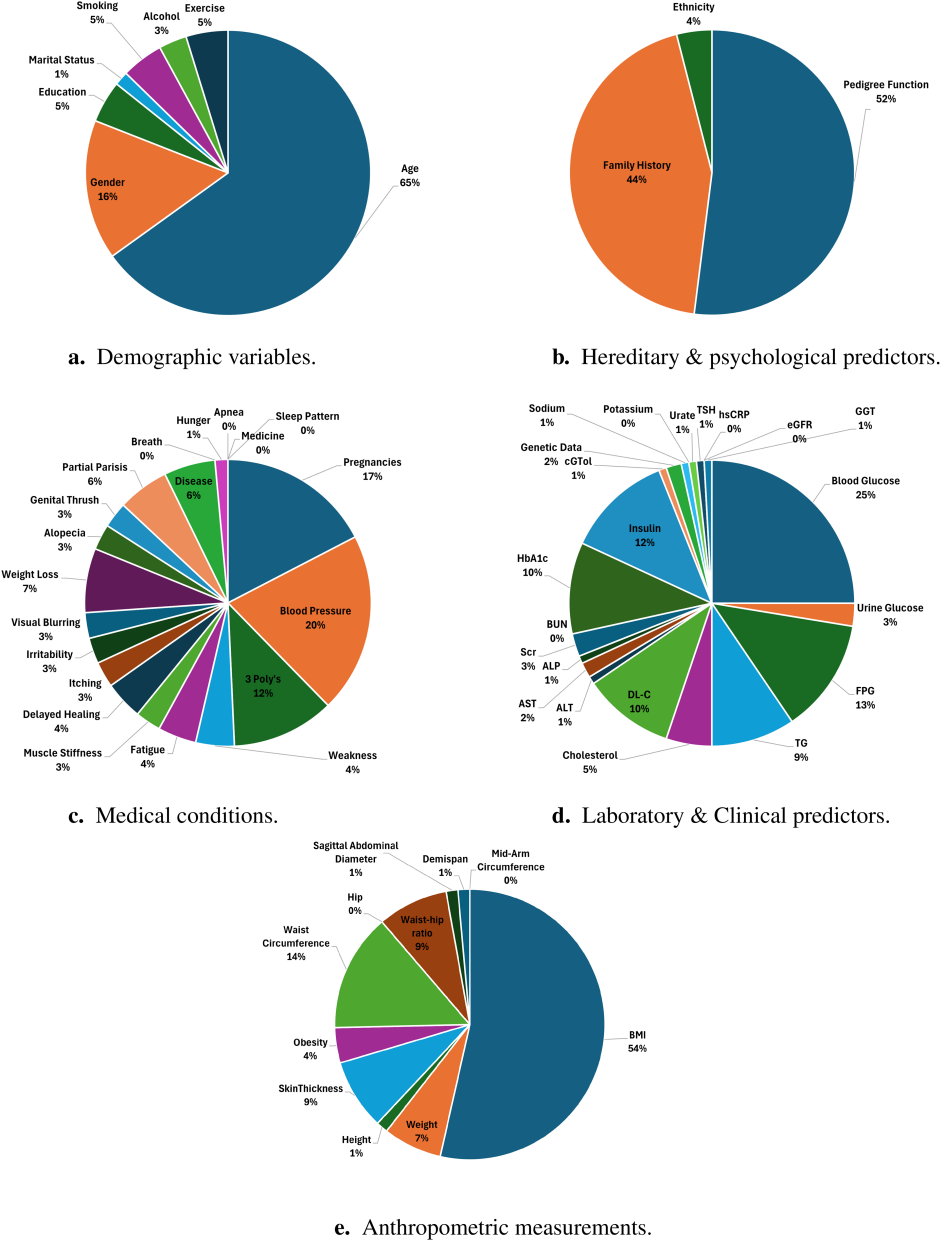
Key predictors of T2DM categorized into demographic, hereditary, medical, laboratory, and anthropometric domains, highlighting their relative significance. (**a**) Demographic variables. (**b**) Hereditary & psychological predictors. (**c**) Medical conditions. (**d**) Laboratory & Clinical predictors. (**e**) Anthropometric measurements.

**Predominant predictors used for training ML models:** From 2021 to 2024, numerous studies investigated various predictors encompassing domains such as demographic, medical condition, hereditary, anthropometric, and laboratory data. To provide clarity, we categorised these predictors into five groups: demographic, medical condition, lifestyle, hereditary & psychological, anthropometric, and laboratory data. Some studies [e.g., (146, 147)] integrated demographic, lifestyle, and clinical data to predict diabetes. Tables 5–9 provide a comprehensive summary of predictors used in T2DM prediction studies, emphasizing their frequency and significance. Predictors marked with^†^ are identified as the most influential by the authors. Figure 6 visualizes the distribution of these key predictors through a pie chart, where each slice represents a significant factor, proportional to its frequency across studies. Each slice’s size reflects how often different studies have identified it as a key determinant of T2DM risk. While the tables present a broad overview of all explored predictors, the pie chart highlights only those deemed most impactful (^†^).

1. **Demographic data & lifestyle:** In predicting T2DM, demographic and lifestyle predictors play a crucial role. Table 5 shows the different features used by various studies. Figure 6a highlights that age and gender are the most influential predictors in the demographic domain, emphasizing their critical role in the development and progression of T2DM. Ageing correlates with the natural decline in insulin sensitivity and beta-cell function, which are critical determinants of T2DM development. Several studies, including (137, 168, 194), emphasize age as a primary determinant. Similarly, gender disparities in T2DM prevalence and progression, influenced by hormonal changes and lifestyle differences, are evident in studies such as (148, 151). Lifestyle factors, including smoking, alcohol consumption, and physical activity, are modifiable predictors with a direct influence on metabolic health. Regular physical activity is shown to significantly lower T2DM risk, while sedentary lifestyles and high alcohol intake exacerbate insulin resistance and hyperglycemia (117, 199). Smoking introduces oxidative stress and inflammation, further increasing diabetes risk. These insights are pivotal for designing public health interventions targeting lifestyle modifications.
2. **Hereditary & psychological:** Hereditary factors, including the pedigree function and family history of diabetes, are robust indicators of genetic predisposition, as shown in Table 6, Figure 6b. These predictors capture the familial aggregation of T2DM and are widely cited in studies such as (163, 166). Psychological factors, including stress and psychiatric disorders, are emerging as significant contributors to T2DM risk. Patients with mental illnesses exhibit higher prevalence rates of T2D due to lifestyle disruptions, medication side effects, and stress-induced physiological changes (137). Machine learning models incorporating these predictors provide enhanced accuracy in risk stratification by capturing the intricate interplay between mental health and metabolic function.
3. **Medical condition:** Medical conditions offer critical insights into the biological and physiological precursors of T2DM, as summarized in Table 7. Figure 6c highlights pregnancies and blood pressure as key predictors in this domain. Pregnancies, particularly those complicated by gestational diabetes, significantly increase the risk of future T2DM, as demonstrated in studies like (122) and (167). Blood pressure, another core component of metabolic syndrome, is strongly associated with insulin resistance and T2DM. This relationship is highlighted in studies by (148, 170). Classic symptoms of diabetes, commonly referred to as the “3 Polys”—polyuria, polydipsia, and polyphagia—are commonly acknowledged as cardinal signs of diabetes. Studies such as (150, 180) emphasise their diagnostic importance. These symptoms, combined with other medical conditions, enhance the sensitivity and specificity of ML models in diagnosing T2DM.
4. **Laboratory/clinical:** Laboratory and clinical markers offer precise, quantifiable data for T2DM prediction. It can be observed from Table 8, Figure 6d that blood glucose levels, insuline and HbA1c are foundational metrics for diagnosing and monitoring T2DM. Studies such as (168, 194) consistently identify these as the most significant laboratory predictors. Lipid profiles, including TG and HDL-C, provide insights into metabolic health (117, 200). High TG levels and low HDL-C are associated with insulin resistance, as demonstrated by (137, 198). Additionally, renal function markers such as Scr and BUN are critical for assessing diabetes-associated kidney complications, as highlighted in (177). Liver function tests, including ALT, AST, and GGT, are increasingly recognised for their role in T2DM risk prediction. Elevated levels of these enzymes often correlate with non-alcoholic fatty liver disease (NAFLD), a condition closely linked to insulin resistance, as reported by (177, 185, 192).
5. **Anthropometric measurements:** Anthropometric measurements are non-invasive, cost-effective predictors of T2DM. Table 9 shows different anthropometric features used by various studies. Anthropometric measurements are critical in evaluating obesity and its role in T2D pathogenesis. It can be observed from Figure 6e that among different features, indicators like BMI, waist circumference, and waist-to-hip ratio are widely regarded as reliable predictors (117, 148, 187, 201). Advanced indices such as body adiposity index and body shape index offer refined assessments of body composition and its metabolic implications (201). These predictors are particularly valuable in population-based screening programs, enabling early identification of at-risk individuals. These findings confirm that diabetes is a multifaceted disease influenced by various factors, necessitating a comprehensive and interdisciplinary approach for its understanding and management.

**Table 5:** Shows the demographic predictors used by the authors.

Demographic data & lifestyle	
Predictors	References
Age	(123, 137, 148)^†^ (149–151)^†^ (146, 152)^†^ (153–155)^†^ (147)^†^ (122, 156)^†^ (157)^†^ (158)^†^ (159)^†^ (160)^†^ (161)^†^ (162)^†^ (163)^†^ (164, 165)^†^ (166, 167)^†^ (169)^†^ (170, 171)^†^ (172)^†^ (173)^†^ (126)^†^ (174)^†^ (175)^†^ (140)^†^ (176)^†^ (177)^†^ (178, 179)^†^ (180–184)^†^ (185)^†^ (186)^†^ (187)^†^ (188)^†^ (189)^†^ (190–192)^†^ (117)^†^ (193)^†^ (125, 194)^†^ (195)^†^ (196)^†^ (197)^†^
Gender	(148–152, 188)^†^ (146)^†^ (155, 156)^†^ (122)^†^ (164, 165)^†^ (140, 170)^†^ (173)^†^ (126)^†^ (174, 175, 177–179)^†^ (190)^†^ (117, 125, 137, 180–182, 184, 186, 187, 189, 192–194)
Education	(146)^†^ (187)^†^ (140)^†^ (192)
Marital status	(140, 146)^†^
Smoking	(123, 140, 148, 189)^†^ (198)^†^ (117, 192, 193)^†^
Alcohol	(123, 140, 148, 189, 198)^†^ (117, 192, 193)^†^
Exercise	(123, 148)^†^ (140, 188, 189)^†^ (198)^†^ (117, 192)

^†^Highlights the factor that the authors believe to have the most notable influence.

**Table 6:** Shows the hereditary & psychological related predictors used by the authors.

Hereditary & psychological	
Predictors	References
Pedigree function	(151, 154, 155)^†^ (157, 158, 160)^†^ (161, 162)^†^ (163)^†^ (166, 167)^†^ (168, 169)^†^ (171)^†^ (172)^†^ (174, 176) (181)^†^ (159, 183)^†^ (193)^†^ (191, 195)^†^ (196)^†^ (197)
Family history	(123)^†^ (148)^†^ (151)^†^ (122)^†^ (161, 164, 188)^†^ (173)^†^ (126)^†^ (175)^†^ (177, 184, 186, 189)^†^ (117)^†^ (193)^†^ (125)
Ethnicity	(151)^†^ (117, 125, 140)

^†^Highlights the factor that the authors believe to have the most notable influence.

**Table 7:** Shows the medical realted predictors used by the authors.

Medical condition	
Predictors	References
Pregnancies	(151, 154, 155)^†^ (157)^†^ (158, 160)^†^ (161, 188)^†^ (162, 163, 167)^†^ (166)^†^ (168)^†^ (169)^†^ (171, 172)^†^ (159, 174, 176, 181, 183, 191)^†^ (195)^†^ (196, 197)^†^
Blood pressure	(137, 151)^†^ (146, 147)^†^ (122)^†^ (161, 163)^†^ (166)^†^ (169, 170)^†^ (174)^†^ (176–178)^†^ (181, 183, 184, 185, 187)^†^ (140, 159, 189)^†^ (198)^†^ (193)^†^ (125)^†^ (194)^†^ (191, 195–197)
3 Poly’s	**Polyuria** (161, 173)^†^ (150)^†^ (152)^†^ (148)^†^ (156)^†^ (165)^†^ (170, 179)^†^ (180)^†^ (193)
	**Polydypsia** (150)^†^ (152)^†^ (156)^†^ (165)^†^ (170, 179)^†^ (180)^†^
	**Polyphagia** (150, 152, 156)^†^ (165)^†^ (170, 179, 180)^†^
Weakness	(150, 152, 156)^†^ (165)^†^ (170, 179, 180)^†^
Muscle stiffness	(150, 152, 156)^†^ (165)^†^ (170, 179, 180)
Delayed healing	(150, 152, 156)^†^ (165)^†^ (148)^†^ (170, 179, 180)
Itching	(150, 152, 156)^†^ (165)^†^ (148, 170, 179, 180)
Irritability	(150, 152, 156)^†^ (165)^†^ (170, 179, 180)
Fatigue	(173)^†^ (148, 193)^†^ (193)^†^
Visual blurring	(150–152, 156)^†^ (165)^†^ (148, 170, 179, 180)
Weight loss	(150, 152, 156)^†^ (122)^†^ (165)^†^ (170, 179)^†^ (180)^†^ (185)
Alopecia	(150, 152, 156)^†^ (165)^†^ (170, 179, 180)
Genital thrush	(150, 152, 156)^†^ (165)^†^ (170, 179, 180)
Partial parisis	(150, 152, 156)^†^ (165)^†^ (170, 179)^†^ (180)^†^
Disease	**Diabetes** (150, 152, 156)^†^ (165, 170)^†^ (179, 180)
	**Cardiovascular** (151)^†^ (137, 153, 189),
	**Liver** (177)
	**Kidney** (177)
	**Frequent infection** (148)
	**Psychlogical disorder** (137, 148, 161)^†^ (194)
Breath	**Breath-rate** (147)
Hunger	(148)^†^
Apnea	(137, 161)
Medicine	(137, 161, 175)
Sleep pattern	(198)

^†^Highlights the factor that the authors believe to have the most notable influence.

**Table 8:** Shows the laboratory & clinical related predictors used by the authors.

Laboratory/clinical	
Predictors	References
Blood glucose	(151, 155)^†^ (154)^†^ (157)^†^ (158)^†^ (160)^†^ (161)^†^ (162)^†^ (163, 167)^†^ (166)^†^ (168)^†^ (169)^†^ (171)^†^ (170)^†^ (172)^†^ (174)^†^ (175)^†^ (176)^†^ (117)^†^ (177)^†^ (181)^†^ (183)^†^ (188)^†^ (159)^†^ (190)^†^ (193)^†^ (196)^†^ (191)^†^ (195)^†^ (197)^†^
Urine glucose	(137)^†^ (147)^†^
FPG	(137)^†^ (148)^†^ (123)^†^ (149)^†^ (153)^†^ (146)^†^ (147, 155)^†^ (126)^†^ (175)^†^ (177, 178, 181, 184)^†^ (185)^†^ (189)^†^ (198)^†^ (194)^†^
TG	(123, 137)^†^ (149)^†^ (117)^†^ (153) (146) (126, 147, 164)^†^ (174, 175)^†^ (148, 177, 178, 184, 185)^†^ (186, 187)^†^ (189, 198) (192)^†^ (194)^†^
DL-C	**HDL-C** (137, 149)^†^ (148)^†^ (123)^†^ (153)^†^ (146, 147, 170)^†^ (174, 177, 190, 198)^†^ (178)^†^ (185, 186)^†^ (192, 198)^†^ (117)^†^ (194)^†^
	**Non-HDL-C** (187)^†^ (189)^†^,
	**LDL-C** (137, 149)^†^ (153)^†^ (146–148, 174, 177, 178, 184)^†^ (189)^†^ (117)^†^ (123, 185, 186, 198)^†^ (192)^†^ (194)^†^
	**VLDL** (148)^†^
ALT	(146, 164, 177, 178, 184, 187)^†^ (137, 192)
AST	(187) (146, 164, 177, 187)^†^ (192)^†^
ALP	(192)^†^
Scr	(146, 147, 155, 174, 177, 178)^†^ (181, 184, 186)^†^ (148, 192)^†^
BUN	(146, 147, 149, 174, 184, 192)
SUA	(126, 146, 151)^†^ (185)
TBIL	(146)
HbA1c	(188)^†^ (123)^†^ (137, 149)^†^ (155, 164)^†^ (126, 174, 177, 181, 185)^†^ (148)^†^ (117)^†^ (186, 187)^†^ (198)^†^ (185)^†^
Insulin	(123)^†^ (151, 154, 155, 157)^†^ (158, 160–163)^†^ (166, 167)^†^ (168)^†^ (169)^†^ (171, 172)^†^ (174)^†^ (176)^†^ (181, 183)^†^ (188)^†^ (159)^†^ (189)^†^ (191, 193, 195, 196)^†^ (197)
cGTol	(164)^†^
Genetic data	(164)^†^ (126)^†^ (202)
Cholestrol	(151)^†^ (146, 147, 153, 164, 170)^†^ (140, 174, 177, 184, 186, 187)^†^ (140)^†^ (192, 198)^†^ (194)^†^
Serum	**Sodium** (125, 155, 181)^†^
	**Potassium** (125, 155, 181)
	**Urate** (117)^†^
TSH	(185, 187)^†^
hsCRP	(137, 164)
Cortisone	(153)
eGFR	(137)
GGT	(117)^†^

^†^Highlights the factor that the authors believe to have the most notable influence. TG, triglycerides; ALT, alanine aminotransferase test; AST, aspartate aminotransferase test; ALP, alkaline phosphatase; BUN, blood urea nitrogen; TBIL, total bilirubin; cGToL, current glucose tolerance status; TSH, thyroid stimulating hormone; eGFR, estimated glomerular filtration rate; Hs-CRP, high-sensitivity C-reactive protein; GGT, Gamma-Glutamyl transferase; TSH, thyroid stimulating hormone.

**Table 9:** Shows the anthropometric measurements related predictors used by the authors.

Anthropometric measurements	
Predictors	References
BMI	(137, 149)^†^ (153–155)^†^ (147)^†^ (157)^†^ (148)^†^ (123)^†^ (158)^†^ (171)^†^ (160)^†^ (161)^†^ (162)^†^ (163)^†^ (164, 167)^†^ (166)^†^ (168, 169)^†^ (170, 172)^†^ (126)^†^ (174, 175)^†^ (176)^†^ (178, 181, 183)^†^ (184)^†^ (185)^†^ (117)^†^ (186, 187)^†^ (188)^†^ (159)^†^ (140)^†^ (190, 198)^†^ (192)^†^ (193)^†^ (125)^†^ (194)^†^ (201)^†^ (195)^†^ (196)^†^ (197)^†^
	**Body Roundness Index** (201)
	**Body Adiposity Index** (201)^†^
	**Body Shape Index** (201)
Weight	(123, 137, 148, 170, 173)^†^ (185)^†^ (153)^†^ (189)^†^ (192, 201)
Height	(123, 153, 170, 173)^†^ (137, 185, 201)
Body size	**Waist** (146)^†^ (122)^†^ (148, 164)^†^ (189)^†^ (170)^†^ (185, 187)^†^ (198)^†^ (117)^†^ (193)^†^ (201)^†^
	**Hip** (170, 187)
	**Waist-hip ratio** (147)^†^ (170)^†^ (117)^†^ (175)^†^ (187)^†^ (192, 193)^†^ (201)
	**Waist-to-Height ratio** (201)
	**Sagittal abdominal diameter** (122)^†^
	**Demispan** (201)^†^
	**Mid-arm circumference** (201)
SkinThickness	(151, 154, 155, 157, 158, 160–163, 188)^†^ (123, 166, 167)^†^ (168)^†^ (169, 171, 172)^†^ (174, 176, 181, 183, 186)^†^ (159, 191, 193, 195, 196)^†^ (197)^†^
Obesity	(150, 151)^†^ (152, 156)^†^ (165, 170, 177)^†^ (179, 180, 189)

^†^Highlights the factor that the authors believe to have the most notable influence.

**Emerging trends:** Researchers are increasingly leveraging ML and AI tools to enhance the accuracy and robustness of predictive models for T2DM. The integration of omics data, including genomics, proteomics, metabolomics, and transcriptomics, is another significant trend (203). By incorporating these comprehensive molecular datasets, researchers aim to uncover the underlying biological mechanisms of T2DM. The use of omics data facilitates the identification of novel biomarkers and enhances the predictive power of models, offering deeper insights into the disease’s aetiology and progression. The use of real-time data from wearable technologies (204, 205) is an emerging trend in diabetes research. Devices such as activity trackers and continuous glucose monitors provide real-time insights into patients’ physical activity, dietary habits, and blood glucose levels. These data are invaluable for developing dynamic prediction models that can adapt to changing health behaviors and conditions, enabling timely interventions. There is a noticeable trend towards integrating data from multiple sources to enhance the robustness of predictive models. For example, combining genetic data from the UK Biobank with lifestyle information from NHANES or EHRs provides a more comprehensive risk assessment (117, 141, 206). The application of ML and AI techniques to these datasets is increasing, with researchers leveraging these advanced analytical tools to develop more accurate and personalized prediction models.

In conclusion, the frequent use of comprehensive datasets such as the UK Biobank, EHRs, and national health surveys underscores their critical role in advancing T2DM prediction research. The integration of multiple data sources, the application of ML, and the focus on personalized and preventive medicine are key trends shaping the future of this field. These efforts aim to improve the accuracy of predictions and the effectiveness of interventions, ultimately contributing to better health outcomes for individuals at risk of T2DM.

## 6 Future directions

Building on the insights from this comprehensive analysis, several future directions are proposed to further advance the field of T2DM prediction research:

1. **Digital Twins (DT) and Real-Time monitoring for personalized diabetes care:** The integration of DTs and real-time monitoring offers a transformative approach to T2DM prediction and management. DTs create virtual replicas of patients using real-time data from wearable devices, CGMs, and EHRs to simulate personalized treatment strategies, predict complications, and optimize disease management (208, 209). Meanwhile, advancements in wearable technology (e.g., smartwatches, biosensors, insulin pumps) enable continuous health monitoring, allowing AI models to detect glucose fluctuations, automate insulin adjustments, and provide lifestyle recommendations (209–211). Future research should focus on enhancing DT models with multi-omics data for greater predictive accuracy (212), ensuring interoperability between real-time monitoring systems and healthcare platforms, and developing ML algorithms capable of processing high-frequency health data while maintaining stability and accuracy (213, 214). Additionally, large-scale clinical trials are necessary to validate the effectiveness of these technologies in real-world diabetes management (131, 215, 216).
2. **Strengthening interdisciplinary collaboration:** Collaboration between data scientists, healthcare professionals, and policymakers is essential to develop technically robust and clinically relevant models for T2DM prediction and management. Effective interdisciplinary partnerships can bridge the gap between ML advancements and real-world clinical application, ensuring that models are not only accurate but also interpretable and actionable for healthcare providers (217, 218). Large-scale, multi-center studies are needed to diversify datasets, enhance model generalizability, and improve applicability across different demographic and geographic populations. Additionally, policymakers must prioritize the development and enforcement of standardized regulations, ethical guidelines, and governance frameworks to address the challenges posed by AI in healthcare (219).
3. **Development of explainable AI models:** Our analysis shows that traditional ML models such as SVM, RF, and deep neural networks are widely used for T2DM prediction, but their black-box nature limits transparency and interpretability in clinical settings. Explainable AI (XAI) addresses this issue by offering techniques to interpret model predictions, identify key decision factors, and assess reliability, thereby enhancing trust among healthcare professionals (220, 221). Future research should focus on developing clinically interpretable AI models using techniques like SHAP (Shapley Additive Explanations), LIME (Local Interpretable Model-Agnostic Explanations), and attention mechanisms to provide meaningful insights into model behavior (133, 134). By integrating transparency and interpretability into AI models, XAI can enhance clinical decision-making and facilitate the wider acceptance of AI-driven T2DM management strategies.
4. **Integration of multi-omics data:** The incorporation of multi-omics data, including genomics, proteomics, metabolomics, and microbiomics, provides deeper insights into the biological mechanisms underlying T2DM (203, 222). By integrating these diverse datasets, predictive models can uncover novel biomarkers, disease pathways, and therapeutic targets, leading to improved risk stratification and precision medicine approaches (223). Multi-omics data facilitate the identification of gene-environment interactions, which play a crucial role in diabetes onset and progression. For instance, integrating various omics data has elucidated mechanisms through which T2DM-associated genetic variations impact disease risk (224). Future research should focus on developing advanced machine learning frameworks capable of efficiently integrating multi-omics data to enhance predictive accuracy while maintaining interpretability and scalability. Recent studies have highlighted the potential of DL based approaches for multi-omics data integration in cancer, suggesting similar methodologies could be beneficial in diabetes research (225).
5. **Cross-population validation in predictive modeling:** Generalizability is a major challenge in T2DM predictive modeling, as most models are developed using data from specific ethnic, genetic, and geographic cohorts, restricting their broader applicability. To ensure fairness, equity, and clinical relevance, models must be validated across diverse populations. Variations in genetics, environmental exposures, socioeconomic factors, and healthcare access play a crucial role in diabetes risk, highlighting the necessity of rigorous external validation across heterogeneous datasets. Without it, predictive models may perpetuate healthcare disparities and limit their real-world effectiveness. A study developed questionnaire-based prediction models for T2DM prevalence and incidence, training them on a white population and validating them across multiple ethnicities, demonstrating the importance of such cross-population validation (226). Future research should prioritize multi-center studies that incorporate genetically and environmentally diverse populations to improve model robustness and fairness (227, 228). Additionally, the integration of transfer learning and domain adaptation techniques could help models learn generalizable patterns and improve performance across heterogeneous datasets (229, 230). Ensuring rigorous external validation is crucial for equitable AI-driven diabetes care and broader clinical adoption.
6. **Policymaker guidelines and support:** To facilitate the integration of ML models into public health initiatives, policymakers must play a central role in resource allocation, regulatory oversight, and ethical governance (217, 219). Supporting pilot programs that test and refine AI-driven diabetes prediction models in real-world clinical and community settings will be crucial to ensuring their clinical utility and scalability (231). Policymakers should establish standardized guidelines for AI adoption in healthcare, focusing on data privacy, security, fairness, and algorithmic bias mitigation to promote safe and equitable AI applications (232, 233). Additionally, investment in publicly accessible datasets and federated learning frameworks can enhance data diversity and model generalizability while preserving patient confidentiality (234). Regulatory frameworks must ensure that AI-driven diabetes prediction models adhere to global health standards (e.g., GDPR, HIPAA, FDA/EMA guidelines) and are transparent, explainable, and ethically deployed (22). Encouraging interdisciplinary collaboration among data scientists, clinicians, regulatory bodies, and patient advocacy groups will be key to developing trustworthy AI-driven healthcare solutions that benefit all populations.
7. **Addressing patient privacy and security concerns in data sharing:** Patient data stored on cloud services is vulnerable to breaches, causing privacy concerns that limit data sharing and hinder research (235, 236). Privacy-preserving solutions, such as blockchain and federated learning, should be implemented to protect patient data and encourage widespread adoption (234, 237, 238). Future research should focus on frameworks integrating these technologies to alleviate privacy concerns, encourage data sharing, and improve the diversity and robustness of datasets, enhancing diabetes prediction and management.
8. **Exploring the role of generative AI:** Generative AI, including Large Language Models (LLMs) and Generative Adversarial Networks (GANs), has emerged as a transformative technology with significant potential in healthcare. In T2DM research, generative AI can be leveraged to synthesize realistic patient data, augmenting limited datasets and improving model generalizability. For instance, GANs can generate synthetic EHRs that preserve patient privacy while enhancing the diversity and size of training datasets (239). LLMs like GPT-4 can assist in clinical decision-making by providing personalized recommendations based on patient history and real-time data (240). Future research should explore the integration of generative AI with predictive models to improve their robustness, scalability, and applicability across diverse populations. Additionally, multi-modal AI approaches, integrating text, images, and structured health records, could enhance prediction accuracy and provide a more holistic understanding of diabetes progression (241). However, ethical considerations, such as ensuring data authenticity and mitigating bias in generated data, must be addressed to fully realize the potential of generative AI in T2DM management (242, 243).
9. **Enhancing pointwise reliability:** Beyond overall model accuracy, an equally crucial aspect is pointwise reliability, which refers to assessing the trustworthiness of each individual prediction before it is used in clinical decision-making. Ensuring pointwise reliability is essential for integrating ML models into real-world healthcare settings, where incorrect predictions can have significant consequences (232, 244). To enhance pointwise reliability, uncertainty quantification techniques should be employed. Bayesian neural networks, for example, estimate uncertainty by treating model parameters as probability distributions rather than fixed values (245), while conformal prediction provides mathematically rigorous confidence intervals for individual predictions (246). Additionally, ML models often produce probability estimates that may not align with real-world likelihoods. Therefore, calibration techniques, such as Platt scaling (247), isotonic regression (248), and temperature scaling (249), should be explored to adjust model outputs and improve their interpretability. Another approach to enhancing reliability is the computation of trust scores, which measure how similar a given patient’s data is to the training distribution, helping clinicians gauge confidence in each prediction (250). Future research should focus on enhancing AI-driven healthcare solutions by integrating out-of-distribution (OOD) detection with a human-in-the-loop approach and developing clinical decision support systems (CDSS) that incorporate confidence scores and reliability indicators. This will enable the identification of novel or unexpected data, ensure expert intervention for uncertain predictions, and provide clinically actionable insights with measurable confidence.

## 7 Conclusion

This study provides a comprehensive bibliometric and literature analysis of ML and AI applications in T2DM prediction over a 33-year period (1991–2024). By analyzing publication trends, thematic clusters, research methodologies, and emerging technologies, we highlight the transformative impact of AI-driven predictive modeling in diabetes research. Our findings indicate a significant shift in research focus, from traditional statistical models in the 1990s to sophisticated ensemble learning and deep learning techniques in recent years. The exponential growth in publications, particularly post-2010, underscores the increasing interest and technological advancements in this domain. However, despite these advancements, several challenges persist. The reliance on a limited number of datasets, lack of model generalizability across diverse populations, and insufficient integration of psychosocial and lifestyle factors hinder the full potential of AI in clinical applications. Moreover, while ML models have shown promising accuracy in T2DM prediction, their adoption in real-world clinical settings remains limited. The increasing use of explainability tools, such as SHAP and LIME, represents a step forward in bridging the gap between AI-driven predictions and clinical decision-making. However, ensuring model interpretability, ethical considerations, and patient-centric outcomes will be crucial for widespread adoption. Future research should prioritize interdisciplinary collaborations, integrating insights from epidemiology, genetics, lifestyle sciences, and computational intelligence. Additionally, efforts should be directed towards developing clinically actionable AI models that enhance early detection, personalized interventions, and ultimately, improved patient outcomes. Addressing these gaps will pave the way for a more effective and equitable application of AI in diabetes prevention and management. By systematically mapping the evolution of ML in T2DM prediction, this study serves as a foundational resource for researchers, clinicians, and policymakers. As AI continues to advance, a collaborative, data-driven, and patient-centered approach will be essential in mitigating the global burden of diabetes and improving healthcare outcomes.

## Footnotes

^1^More details on the methodology can be found in (45).

## Appendix

### Refinement with TF-IDF algorithm

After establishing a foundational set of keywords, further refinement was achieved by applying the TF-IDF algorithm to the preliminary keyword screening dataset. TF-IDF assigns weights to terms based on how frequently they appear in a document and how rare they are across the entire corpus. It is calculated by dividing the number of times a term (t) appears in a document by the total number of terms in the document (d).

Mathematically, TF(t,d) and IDF(t,D) is calculated as follows:

(A1) $$TF(t,d)=\frac{\text{No. of times term }t\text{ appears in doc }d}{\text{Total no. of terms}\;t\;\text{in doc }d}$$

As shown in Equation (A1) Term Frequency (TF) measures the occurrence of a term within a document, whereas Inverse Document Frequency (IDF) adjusts for how common or rare the term is across the entire dataset [Equation (A2)].

(A2) $$IDF(t,D)=\log\left(\frac{\text{Total no. of docs in corpus}\;N}{\text{No. of doc containing term }t+1}\right)$$

Where, t is the term, D is the corpus of documents and N is the total number of documents in the corpus. Finally, the TF-IDF score for a term in a document is calculated by multiplying its TF by its Inverse IDF, as defined in Equation (A3).

(A3) TF-IDF(t,d,D)=TF(t,d)×IDF(t,D)

The idea is that if a term appears multiple times in a document, it is likely to be more important. Whereas, IDF measures how unique or rare a term is across all documents in the corpus. Terms that occur frequently in many documents are considered less important compared to those that occur in only a few documents. IDF is calculated by taking the logarithm of the ratio of the total number of documents to the number of documents containing the term and adding 1 to avoid division by zero errors. In Table A1, the top thirty terms are presented, ranked by their TF-IDF scores, which highlight those with the greatest impact on specialized discussions within the field.

**Table A1:** Top TF-IDF score keyword.

Word	TF	IDF	TF-IDF	Word	TF	IDF	TF-IDF
Risk factor	385.00	2.71	1.00	Neural network	784.00	2.23	0.80
Diabetes prediction	641.00	1.91	1.00	Diabetes risk	125.00	3.35	0.79
Data mining	438.00	2.66	0.99	Diabetes patient	157.00	3.27	0.78
Risk prediction	210.00	3.19	0.97	Predictive model	180.00	3.19	0.72
Risk score	100.00	4.37	0.97	Gradient boosting	125.00	3.56	0.72
Diabetes mellitus	1421.00	1.56	0.95	diabetes dataset	156.00	3.03	0.71
Type 2 diabetes	1315.00	1.75	0.93	Artificial intelligence	108.00	3.99	0.70
Machine learning	2301.00	1.36	0.93	Deep neural	109.00	3.46	0.69
Logistic regression	457.00	2.35	0.90	Learning method	148.00	3.14	0.68
Prediction model	276.00	3.06	0.87	Diabetes disease	113.00	3.49	0.67
Deep learning	503.00	2.33	0.86	Type 2 diabetes mellitus	119.00	3.30	0.67
Risk assessment	357.00	3.07	0.84	Nearest neighbor	200.00	3.08	0.63
Decision tree	122.00	3.52	0.83	Prediction diabetes	218.00	2.70	0.63
Random forest	483.00	2.30	0.83	Learning model	275.00	2.77	0.63
Learning algorithm	481.00	2.26	0.81	Machine learning approach	98.00	3.58	0.62
